# Supplementary material for: Metaproteomics reveals enzymatic strategies deployed by anaerobic microbiomes to maintain lignocellulose deconstruction at high solids
Source: Nat Commun. 2022 Jul 5;13:3870. doi: 10.1038/s41467-022-31433-x (PMC9256739; doi:10.1038/s41467-022-31433-x)
Supplement: Supplementary file 1 — Supplementary Information [file 41467_2022_31433_MOESM1_ESM.pdf]

Metaproteomics reveals enzymatic strategies deployed by anaerobic microbiomes to maintain lignocellulose deconstruction at high solids

Chirania *et al*.

Supplementary Table 1. The increasing solids loading reactor operating-time length and steady state-time length at each condition at a 10 day residence time. The data for 30 g/L was published prior in Liang *et al.* [1].

Solids loading (g/L)	Total operation per solids loading (days)	Steady state duration (days)
30	50	20
75	204	44
120	85	55
150	217	187

Supplementary Table 2. Reactor steady state data summary. Results are expressed as mean ± one standard deviation. The data for 30 g/L was published prior in Liang *et al*. [1].

**Solids loading solids (g/L)**	**Mean ± standard deviation (g/L)**		**Number of samples**
	Fractional carbohydrate solubilization		
30	0.664±0.026		7
75	0.670±0.004		6
120	0.652±0.011		8
150	0.686±0.033		11
	Biogas production rate (mL/day)		
30	851.3±65.5		10
75	2703.3±77.1		44
120	4294.1±261.7		53
150	6030.1±481.2		187
	Biogas composition		
	CH_4 (%)	CO_2 (%)	
30	49.1 ±1.3	47.2 ±1.3	10
75	49.9±1.5	48.0±1.8	12
120	49.6±1.1	49.0±1.3	17
150	48.7±1.0	48.8±1.3	31
	Volatile fatty acids (g/L Propionic acid)		
30	0.0±0.0		7
75	0.0±0.0		6
120	0.035±0.031		8
150	0.004±0.010		15
	Electron balance		
30	89.3%		1
75	98.8±1.7%		2
120	107.0%		1
150	98.7±2.2%		4
	Mass balance		
30	95.8%		1
75	103.8±0.3%		3
120	102.6±2.5%		3
150	106.2±1.4%		3
	Remaining solids concentration (g/L)		
30	16.4±0.361 g/L		7
75	38.8±0.356		3
120	59.6±1.03		3
150	72.9±2.81		3

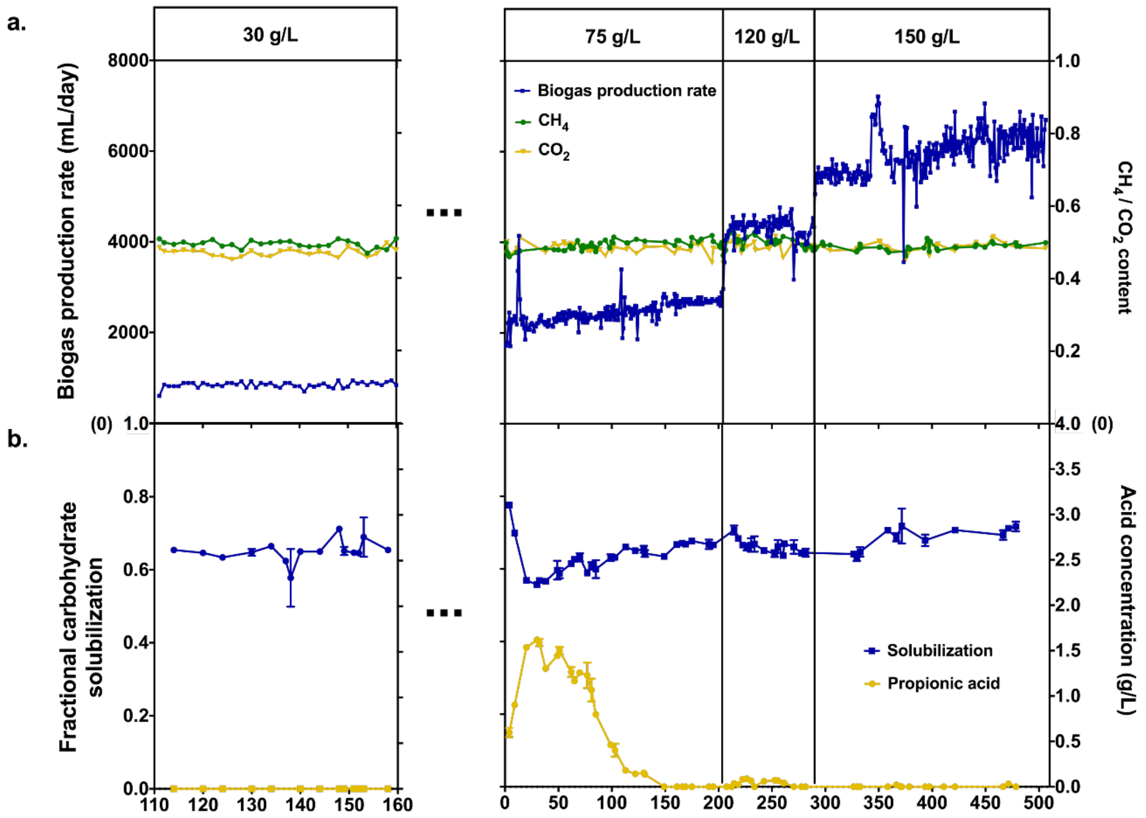

Supplementary Figure 1. Microbiome performance at increasing solids loadings shown by time-course measurements (residence time=10 days). (a) Biogas production rate (mL/day) and composition (CH_4 and CO_2 content) vs. time; **(b)** Fractional carbohydrate solubilization (FCS) and volatile fatty acids concentration (g/L) vs. time. The data for 30g/L solids loading was published in Liang et al.[1], the data for increasing solids loadings of 75 g/L, 120 g/L and 150 g/L was collected did not occur in Liang et al, 2018[1]. Each solids loading period is divided by solid black vertical lines. Between 30 g/L and 75 g/L solids loading in the feed at a residence time of 10 days the residence time was further decreased, before returning to 10 day RT, therefore ellipsis was used between 30 g/L and other solids loadings to indicate non continuous data. Biogas production was recorded once per day. Biogas composition is shown by fractional content of CH_4 and CO_2. FCS data were calculated based on total carbohydrates loaded and total residual carbohydrate in each sample. Acid concentration and FCS results are expressed as mean ± one standard deviation.

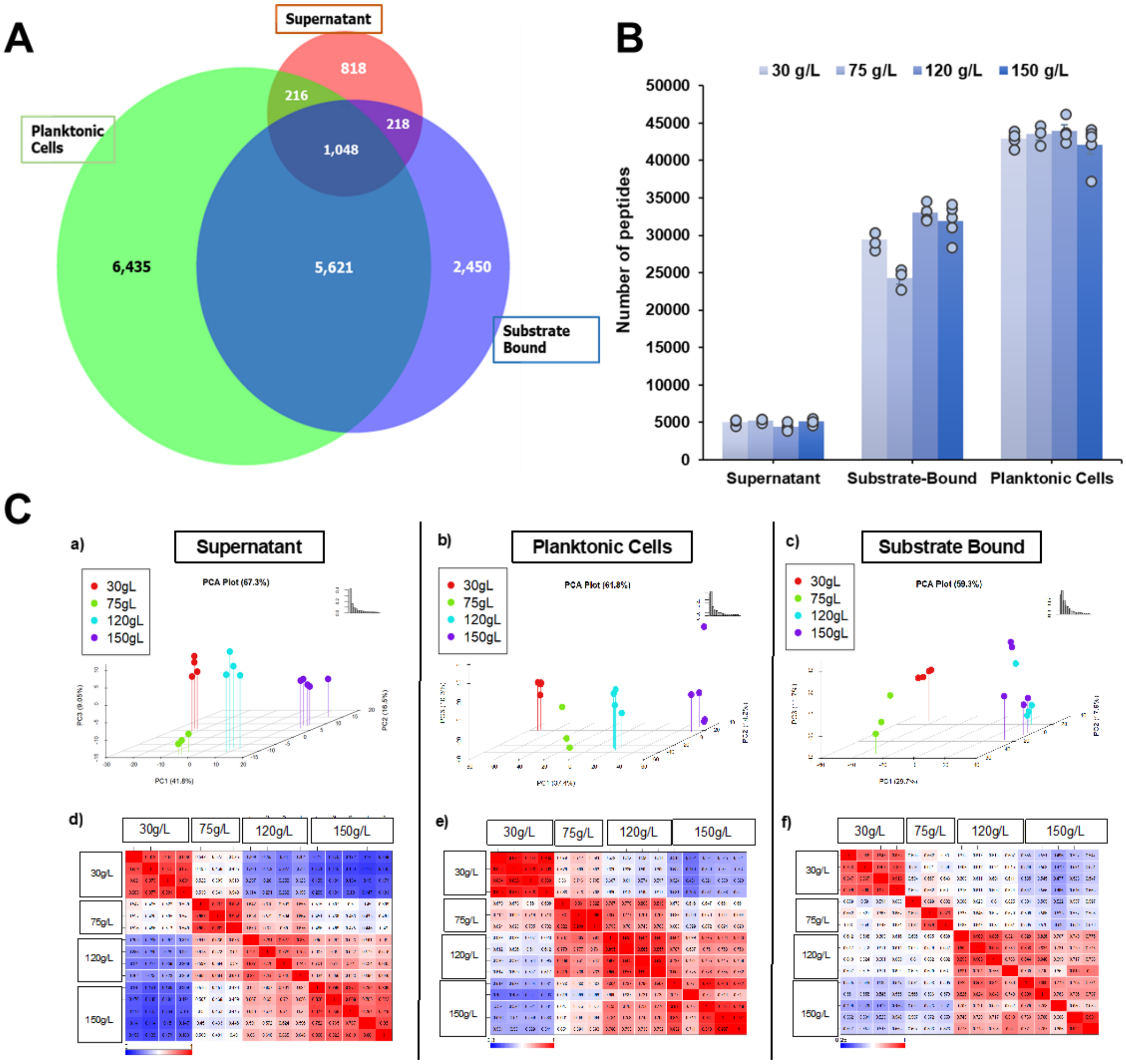

Supplementary Figure 2. Metaproteome metrics and distribution of measured proteins in each fraction. (**A**) Venn diagram shows the overlap of quantifiable proteins across the fractions. (**B**) Count of distinct peptides measured across the solids loadings in each fraction. Error bars show ±standard error of mean (n=4 for 30 and 120 g/L, n=3 for 75 g/L, and n=5 for 150 g/L). (**C**) Top half (a-c) PCA plots of the replicates in each solids loading condition in each fraction. The colors mark the switchgrass substrate loading. Bottom half (d-f) Correlation matrices showing Pearson correlation within the replicates of a solids loading and among the different solids loading conditions in the different fractions. To note, the color scales from low to high correlation are different in the fractions to bring out the contrasts. Source data are provided as a Source Data file.

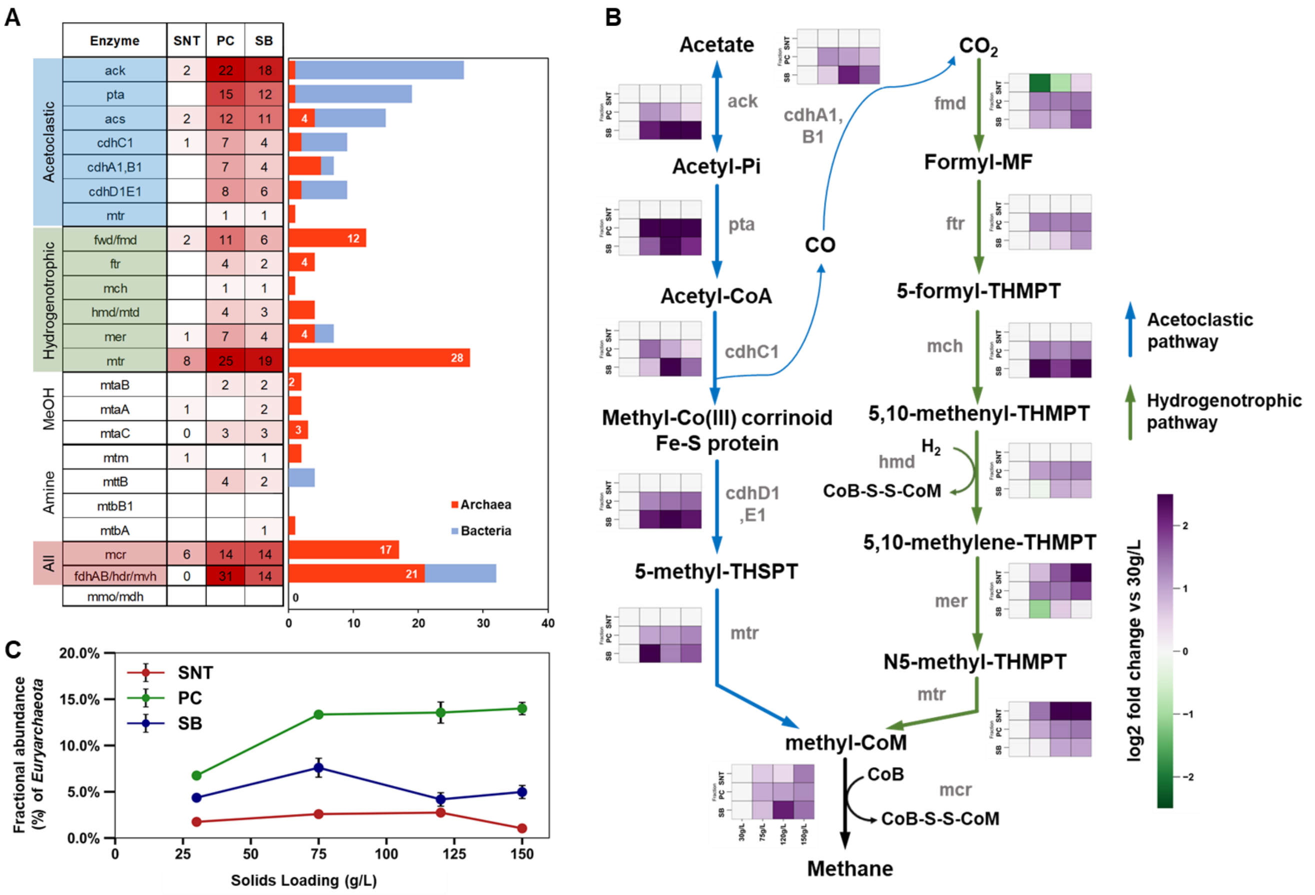

Supplementary Figure 3. Methanogenesis occurs primarily via the hydrogenotrophic pathway. (A) Enumeration of enzymes from methanogenesis pathways quantified in each fraction (darker shade of red scales with quantified proteins for the pathway enzyme) along with their taxonomic origin- Bacterial (blue) and Archaeal (orange). **(B)** Metaproteomics results mapped on the prevalent methanogenesis pathways in the system- acetoclastic and hydrogenotrophic. The heatmap for each reaction in the pathways shows the aggregate abundance trends (log_2 fold difference from 30 g/L) of respective Archaeal enzymes in each fraction. **(C)** The relative contribution of *Euryarchaeota* proteins to the total metaproteome intensity in each fraction. Data are presented as mean values ±SEM (n=4 for 30 and 120 g/L, n=3 for 75 g/L, and n=5 for 150 g/L). SNT: Supernatant, PC: Planktonic cells, and SB: Substrate bound fraction. Source data are provided as a Source Data file.

ack, acetate kinase; **pta**, phosphotransacetylase; **cdh**, acetyl-CoA decarbonylase/synthase; **mtr** (acetoclastic), methyl- H_4PST:CoA methyltransferase; **fwd/fmd**, formylmethanofuran dehydrogenase; **ftr**, formylmethanofuran:H_4MPT formyltransferase; **mch**, methenyl-H_4MPT cyclohydrolase; **hmd**, H_2-forming methylene-H_4MPT dehydrogenase; **mer**, F_{420}-dependent methylene-H_4MPT reductase; **mtr** (hydrogenotrophic), methyl-H_4MPT:coenzyme M methyltransferase; **mtaB**, methanol-5-hydroxybenzimidazolylcobamide Co-methyltransferase; **mtaA**, methanol-CoM methyltransferase; **mtbA**, methylated [methylamine-specific corrinoid protein]:coenzyme M methyltransferase; **mtmB**, methylamine--corrinoid protein Co-methyltransferase; **mttB**, trimethylamine--corrinoid protein Co-methyltransferase; **mtbB1**, dimethylamine--[corrinoid protein] Co-methyltransferase; **mcr**, methyl-coenzyme M reductase; **mmo/mdh**, methane monooxygenase/ methanol dehydrogenase; **THSPT**, tetrahydrosarcinapterin; **THMPT**, tetrahydromethanopterin.

Supernatant fraction: All proteins

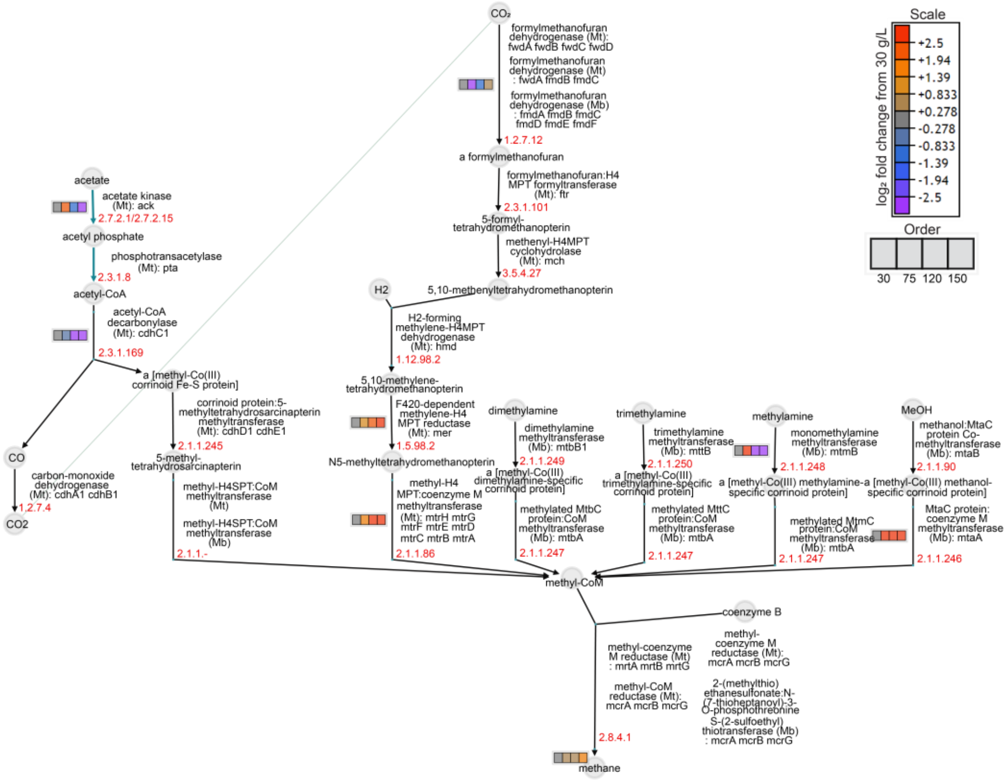

Supernatant fraction: only Archaeal proteins

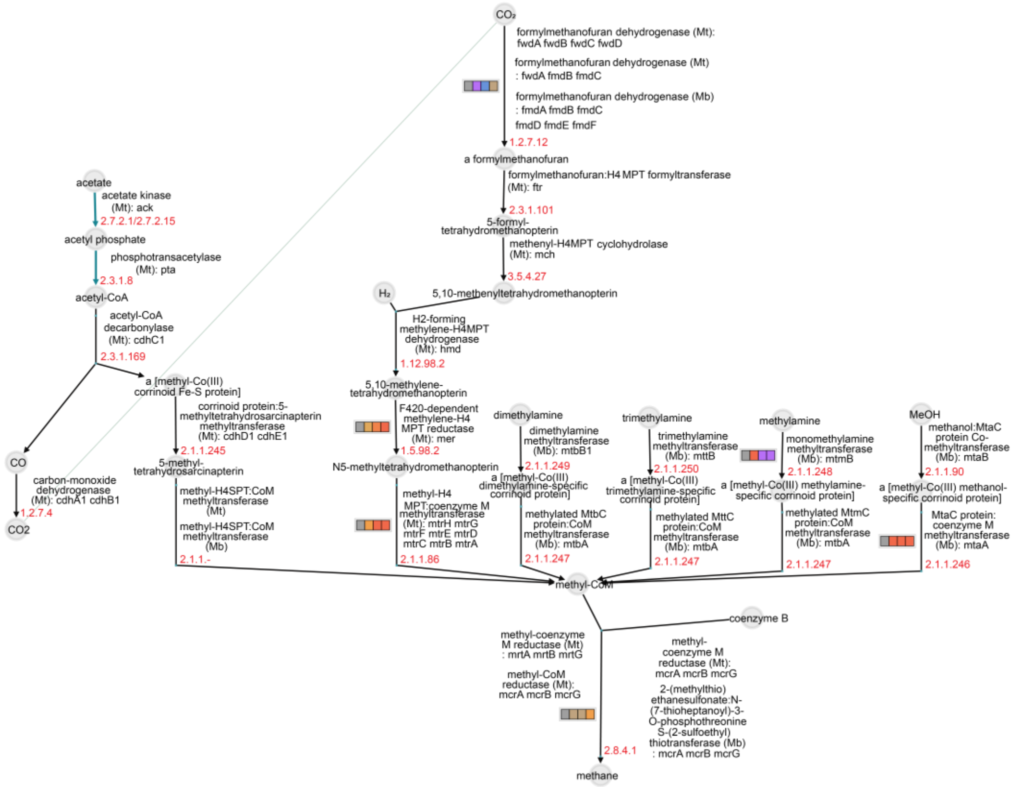

Supplementary Figure 4. **All methanogenesis pathways for the supernatant (SNT**) **fraction.** All identified proteins and proteins from only Archaea were mapped to these pathways. Presence of a heatmap ("heat block") beside an enzyme indicates presence, and the colors indicate fold change in log_2 units from 30 g/L. Order of the heatmap boxes – 30 g/L-75 g/L-120 g/L-150 g/L. Source data are provided as a Source Data file.

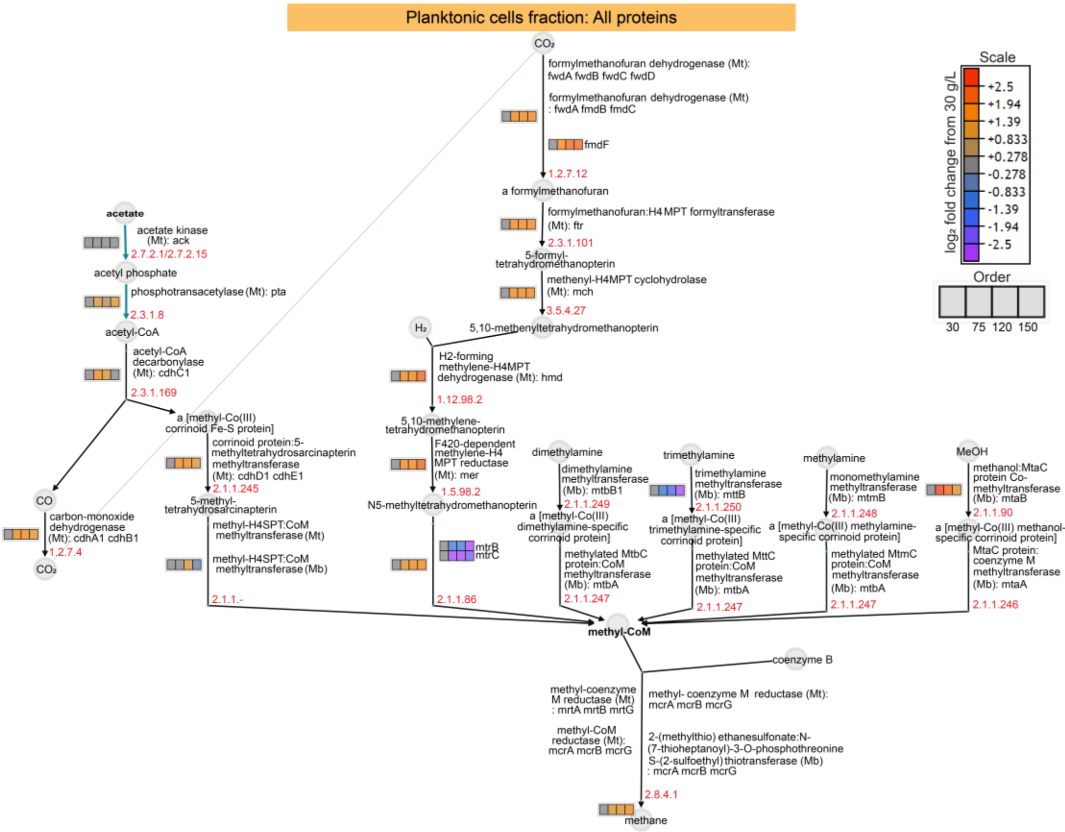
Planktonic cells fraction: All proteins
Scale
log2 fold change from 30 g/L
+2.5
+1.94
+1.39
+0.833
+0.278
-0.278
-0.833
-1.39
-1.94
-2.5
Order
30 75 120 150
CO2
formylmethanofuran dehydrogenase (Mt): fwdA fwdB fwdC fwdD
formylmethanofuran dehydrogenase (Mt) : fwdA fmdB fmdC
fmdF
1.2.7.12
a formylmethanofuran
formylmethanofuran:H4 MPT formyltransferase (Mt): ftr
2.3.1.101
5-formyl-tetrahydromethanopterin
methenyl-H4MPT cyclohydrolase (Mt): mch
3.5.4.27
H2
5,10-methenyltetrahydromethanopterin
H2-forming methylene-H4MPT dehydrogenase (Mt): hmd
1.12.98.2
5,10-methylene-tetrahydromethanopterin
F420-dependent methylene-H4 MPT reductase (Mt): mer
1.5.98.2
N5-methyltetrahydromethanopterin
mtrB
mtrC
2.1.1.86
acetate
acetate kinase (Mt): ack
2.7.2.1/2.7.2.15
acetyl phosphate
phosphotransacetylase (Mt): pta
2.3.1.8
acetyl-CoA
acetyl-CoA decarbonylase (Mt): cdhC1
2.3.1.169
a [methyl-Co(III) corrinoid Fe-S protein]
corrinoid protein:5-methyltetrahydrosarcinapterin methyltransferase (Mt): cdhD1 cdhE1
2.1.1.245
5-methyl-tetrahydrosarcinapterin
methyl-H4SPT:CoM methyltransferase (Mt)
methyl-H4SPT:CoM methyltransferase (Mb)
2.1.1.-
CO
carbon-monoxide dehydrogenase (Mt): cdhA1 cdhB1
1.2.7.4
CO2
dimethylamine
dimethylamine methyltransferase (Mb): mtbB1
2.1.1.249
a [methyl-Co(III) dimethylamine-specific corrinoid protein]
methylated MtbC protein:CoM methyltransferase (Mb): mtbA
2.1.1.247
trimethylamine
trimethylamine methyltransferase (Mb): mttB
2.1.1.250
a [methyl-Co(III) trimethylamine-specific corrinoid protein]
methylated MttC protein:CoM methyltransferase (Mb): mtbA
2.1.1.247
methylamine
monomethylamine methyltransferase (Mb): mtmB
2.1.1.248
a [methyl-Co(III) methylamine-specific corrinoid protein]
methylated MtmC protein:CoM methyltransferase (Mb): mtbA
2.1.1.247
MeOH
methanol:MtaC protein Co-methyltransferase (Mb): mtaB
2.1.1.90
a [methyl-Co(III) methanol-specific corrinoid protein]
MtaC protein: coenzyme M methyltransferase (Mb): mtaA
2.1.1.246
methyl-CoM
coenzyme B
methyl-coenzyme M reductase (Mt) : mrtA mrtB mrtG
methyl-CoM reductase (Mt): mcrA mcrB mcrG
methyl- coenzyme M reductase (Mt): mcrA mcrB mcrG
2-(methylthio) ethanesulfonate:N-(7-thioheptanoyl)-3-O-phosphothreonine S-(2-sulfoethyl) thiotransferase (Mb) : mcrA mcrB mcrG
2.8.4.1
methane

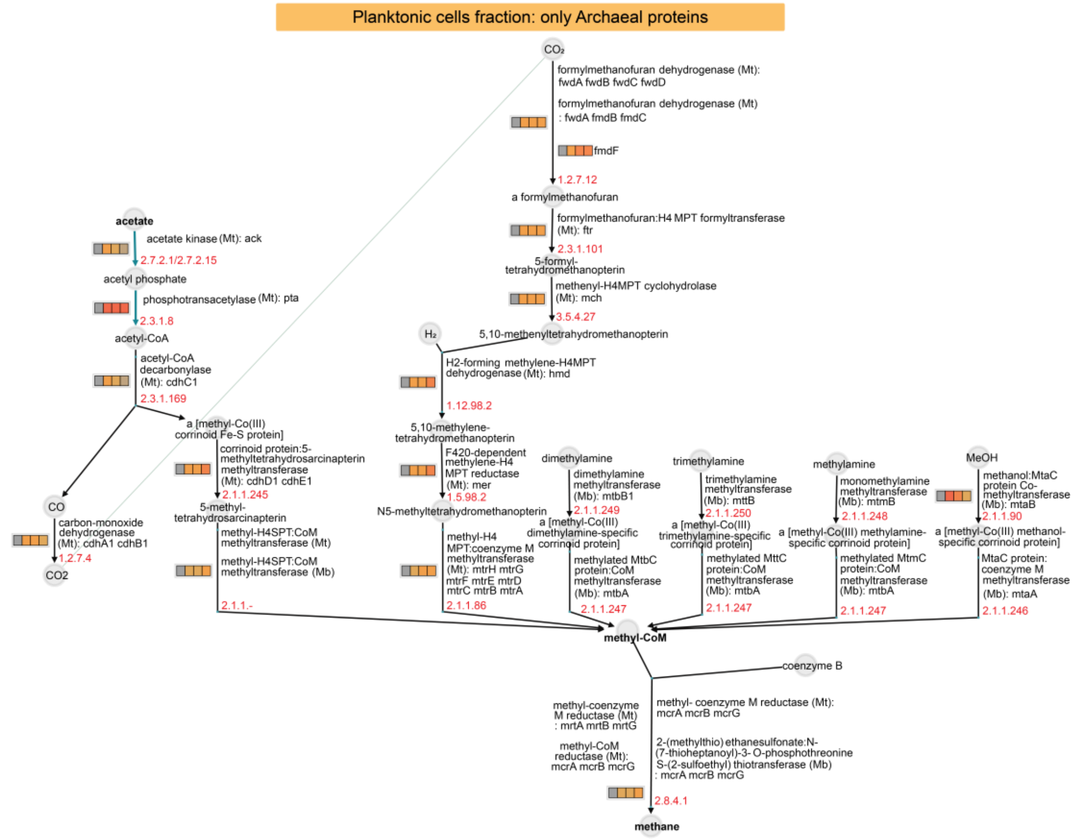
Planktonic cells fraction: only Archaeal proteins
CO2
formylmethanofuran dehydrogenase (Mt): fwdA fwdB fwdC fwdD
formylmethanofuran dehydrogenase (Mt) : fwdA fmdB fmdC
fmdF
1.2.7.12
a formylmethanofuran
formylmethanofuran:H4 MPT formyltransferase (Mt): ftr
2.3.1.101
5-formyl-tetrahydromethanopterin
methenyl-H4MPT cyclohydrolase (Mt): mch
3.5.4.27
H2
5,10-methenyltetrahydromethanopterin
H2-forming methylene-H4MPT dehydrogenase (Mt): hmd
1.12.98.2
5,10-methylene-tetrahydromethanopterin
F420-dependent methylene-H4 MPT reductase (Mt): mer
1.5.98.2
N5-methyltetrahydromethanopterin
methyl-H4 MPT:coenzyme M methyltransferase (Mt): mtrH mtrG mtrF mtrE mtrD mtrC mtrB mtrA
2.1.1.86
acetate
acetate kinase (Mt): ack
2.7.2.1/2.7.2.15
acetyl phosphate
phosphotransacetylase (Mt): pta
2.3.1.8
acetyl-CoA
acetyl-CoA decarbonylase (Mt): cdhC1
2.3.1.169
a [methyl-Co(III) corrinoid Fe-S protein]
corrinoid protein:5-methyltetrahydrosarcinapterin methyltransferase (Mt): cdhD1 cdhE1
2.1.1.245
5-methyl-tetrahydrosarcinapterin
methyl-H4SPT:CoM methyltransferase (Mt)
methyl-H4SPT:CoM methyltransferase (Mb)
2.1.1.-
CO
carbon-monoxide dehydrogenase (Mt): cdhA1 cdhB1
1.2.7.4
CO2
dimethylamine
dimethylamine methyltransferase (Mb): mtbB1
2.1.1.249
a [methyl-Co(III) dimethylamine-specific corrinoid protein]
methylated MtbC protein:CoM methyltransferase (Mb): mtbA
2.1.1.247
trimethylamine
trimethylamine methyltransferase (Mb): mttB
2.1.1.250
a [methyl-Co(III) trimethylamine-specific corrinoid protein]
methylated MttC protein:CoM methyltransferase (Mb): mtbA
2.1.1.247
methylamine
monomethylamine methyltransferase (Mb): mtmB
2.1.1.248
a [methyl-Co(III) methylamine-specific corrinoid protein]
methylated MtmC protein:CoM methyltransferase (Mb): mtbA
2.1.1.247
MeOH
methanol:MtaC protein Co-methyltransferase (Mb): mtaB
2.1.1.90
a [methyl-Co(III) methanol-specific corrinoid protein]
MtaC protein: coenzyme M methyltransferase (Mb): mtaA
2.1.1.246
methyl-CoM
coenzyme B
methyl-coenzyme M reductase (Mt) : mrtA mrtB mrtG
methyl-CoM reductase (Mt): mcrA mcrB mcrG
methyl- coenzyme M reductase (Mt): mcrA mcrB mcrG
2-(methylthio) ethanesulfonate:N-(7-thioheptanoyl)-3- O-phosphothreonine S-(2-sulfoethyl) thiotransferase (Mb) : mcrA mcrB mcrG
2.8.4.1
methane

Supplementary Figure 5. **All methanogenesis pathways for the planktonic cells fraction.** All identified proteins and proteins from only Archaea were mapped to these pathways. Presence of a heatmap (“heat block”) beside an enzyme indicates presence, and the colors indicate fold change in log_2 units from 30 g/L. Order of the heatmap boxes – 30 g/L-75 g/L-120 g/L-150 g/L. Source data are provided as a Source Data file.

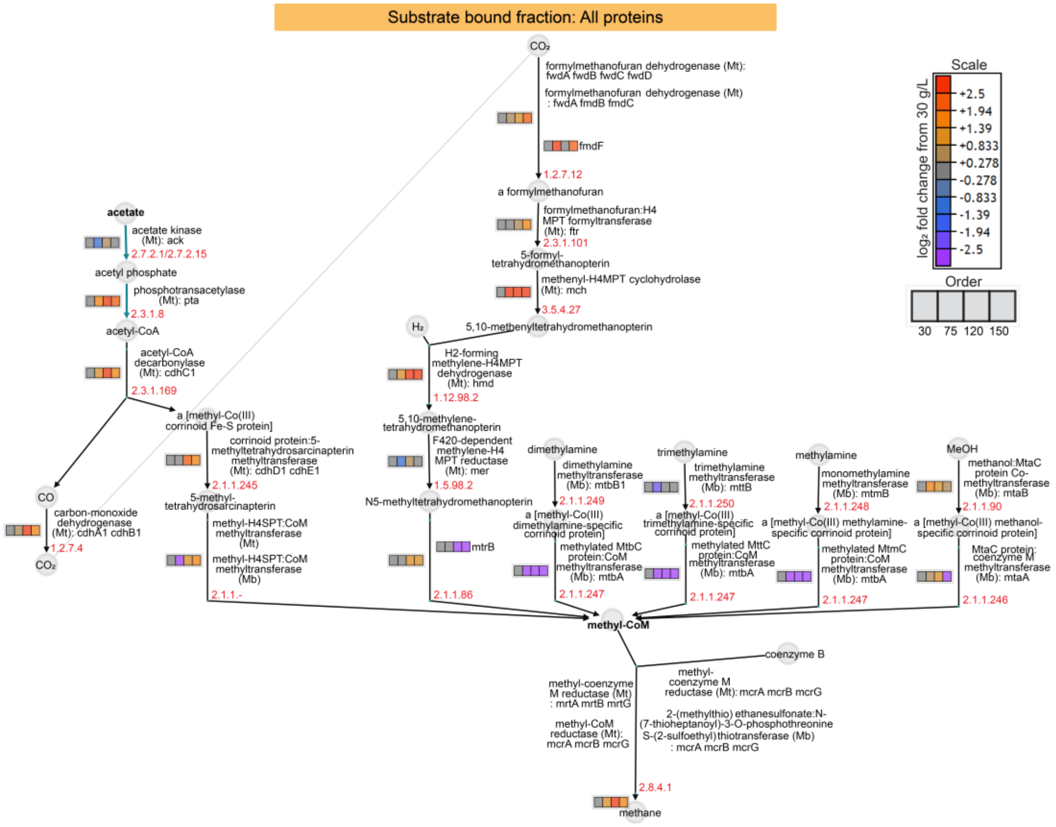
Substrate bound fraction: All proteins
CO₂
formylmethanofuran dehydrogenase (Mt): fwdA fwdB fwdC fwdD
formylmethanofuran dehydrogenase (Mt) : fwdA fmdB fmdC
fmdF
1.2.7.12
a formylmethanofuran
formylmethanofuran:H4 MPT formyltransferase (Mt): ftr
2.3.1.101
5-formyl-tetrahydromethanopterin
methenyl-H4MPT cyclohydrolase (Mt): mch
3.5.4.27
H₂
5,10-methenyltetrahydromethanopterin
H2-forming methylene-H4MPT dehydrogenase (Mt): hmd
1.12.98.2
5,10-methylene-tetrahydromethanopterin
F420-dependent methylene-H4 MPT reductase (Mt): mer
1.5.98.2
N5-methyltetrahydromethanopterin
mtrB
2.1.1.86
acetate
acetate kinase (Mt): ack
2.7.2.1/2.7.2.15
acetyl phosphate
phosphotransacetylase (Mt): pta
2.3.1.8
acetyl-CoA
acetyl-CoA decarbonylase (Mt): cdhC1
2.3.1.169
a [methyl-Co(III) corrinoid Fe-S protein]
corrinoid protein:5-methyltetrahydrosarcinapterin methyltransferase (Mt): cdhD1 cdhE1
2.1.1.245
5-methyl-tetrahydrosarcinapterin
methyl-H4SPT:CoM methyltransferase (Mt)
methyl-H4SPT:CoM methyltransferase (Mb)
2.1.1.-
CO
carbon-monoxide dehydrogenase (Mt): cdhA1 cdhB1
1.2.7.4
CO₂
dimethylamine
dimethylamine methyltransferase (Mb): mtbB1
2.1.1.249
a [methyl-Co(III) dimethylamine-specific corrinoid protein]
methylated MtbC protein:CoM methyltransferase (Mb): mtbA
2.1.1.247
trimethylamine
trimethylamine methyltransferase (Mb): mttB
2.1.1.250
a [methyl-Co(III) trimethylamine-specific corrinoid protein]
methylated MttC protein:CoM methyltransferase (Mb): mtbA
2.1.1.247
methylamine
monomethylamine methyltransferase (Mb): mtmB
2.1.1.248
a [methyl-Co(III) methylamine-specific corrinoid protein]
methylated MtmC protein:CoM methyltransferase (Mb): mtbA
2.1.1.247
MeOH
methanol:MtaC protein Co-methyltransferase (Mb): mtaB
2.1.1.90
a [methyl-Co(III) methanol-specific corrinoid protein]
MtaC protein: coenzyme M methyltransferase (Mb): mtaA
2.1.1.246
methyl-CoM
coenzyme B
methyl-coenzyme M reductase (Mt) : mrtA mrtB mrtG
methyl-coenzyme M reductase (Mt): mcrA mcrB mcrG
methyl-CoM reductase (Mt): mcrA mcrB mcrG
2-(methylthio) ethanesulfonate:N-(7-thioheptanoyl)-3-O-phosphothreonine S-(2-sulfoethyl)thiotransferase (Mb) : mcrA mcrB mcrG
2.8.4.1
methane
Scale
log₂ fold change from 30 g/L
+2.5
+1.94
+1.39
+0.833
+0.278
-0.278
-0.833
-1.39
-1.94
-2.5
Order
30 75 120 150

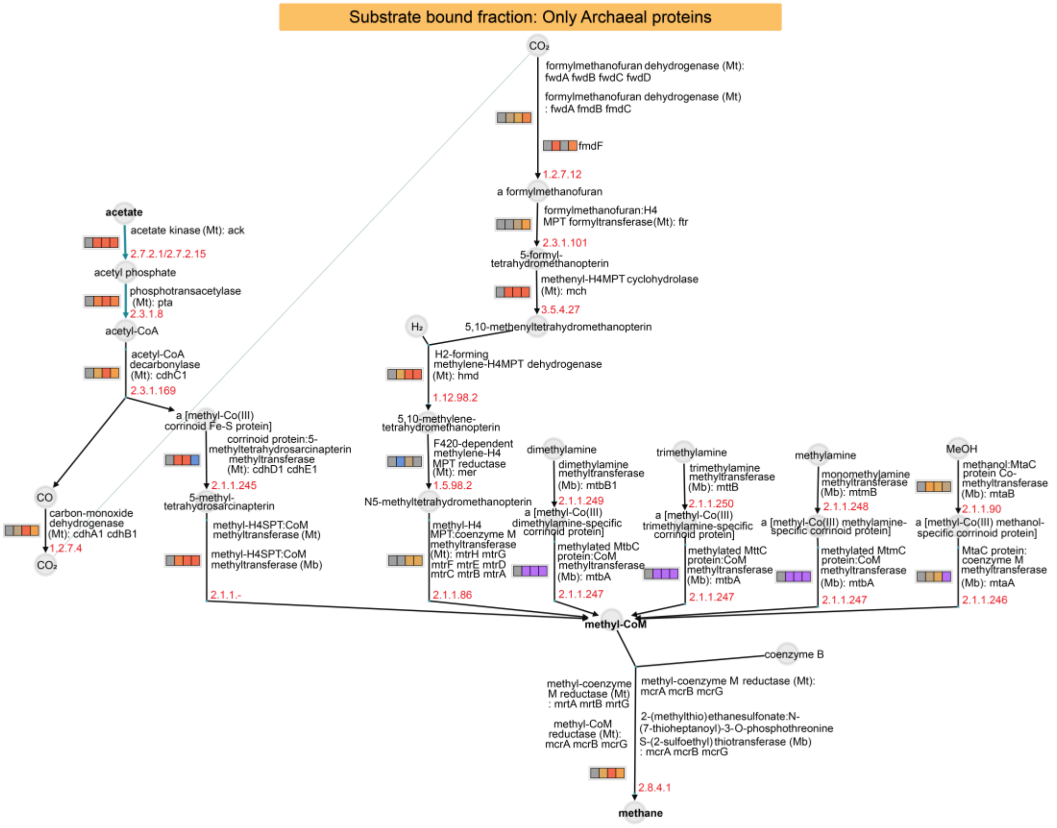
Substrate bound fraction: Only Archaeal proteins
CO₂
formylmethanofuran dehydrogenase (Mt): fwdA fwdB fwdC fwdD
formylmethanofuran dehydrogenase (Mt) : fwdA fmdB fmdC
fmdF
1.2.7.12
a formylmethanofuran
formylmethanofuran:H4 MPT formyltransferase(Mt): ftr
2.3.1.101
5-formyl-tetrahydromethanopterin
methenyl-H4MPT cyclohydrolase (Mt): mch
3.5.4.27
H₂
5,10-methenyltetrahydromethanopterin
H2-forming methylene-H4MPT dehydrogenase (Mt): hmd
1.12.98.2
5,10-methylene-tetrahydromethanopterin
F420-dependent methylene-H4 MPT reductase (Mt): mer
1.5.98.2
N5-methyltetrahydromethanopterin
methyl-H4 MPT:coenzyme M methyltransferase (Mt): mtrH mtrG mtrF mtrE mtrD mtrC mtrB mtrA
2.1.1.86
acetate
acetate kinase (Mt): ack
2.7.2.1/2.7.2.15
acetyl phosphate
phosphotransacetylase (Mt): pta
2.3.1.8
acetyl-CoA
acetyl-CoA decarbonylase (Mt): cdhC1
2.3.1.169
a [methyl-Co(III) corrinoid Fe-S protein]
corrinoid protein:5-methyltetrahydrosarcinapterin methyltransferase (Mt): cdhD1 cdhE1
2.1.1.245
5-methyl-tetrahydrosarcinapterin
methyl-H4SPT:CoM methyltransferase (Mt)
methyl-H4SPT:CoM methyltransferase (Mb)
2.1.1.-
CO
carbon-monoxide dehydrogenase (Mt): cdhA1 cdhB1
1.2.7.4
CO₂
dimethylamine
dimethylamine methyltransferase (Mb): mtbB1
2.1.1.249
a [methyl-Co(III) dimethylamine-specific corrinoid protein]
methylated MtbC protein:CoM methyltransferase (Mb): mtbA
2.1.1.247
trimethylamine
trimethylamine methyltransferase (Mb): mttB
2.1.1.250
a [methyl-Co(III) trimethylamine-specific corrinoid protein]
methylated MttC protein:CoM methyltransferase (Mb): mtbA
2.1.1.247
methylamine
monomethylamine methyltransferase (Mb): mtmB
2.1.1.248
a [methyl-Co(III) methylamine-specific corrinoid protein]
methylated MtmC protein:CoM methyltransferase (Mb): mtbA
2.1.1.247
MeOH
methanol:MtaC protein Co-methyltransferase (Mb): mtaB
2.1.1.90
a [methyl-Co(III) methanol-specific corrinoid protein]
MtaC protein: coenzyme M methyltransferase (Mb): mtaA
2.1.1.246
methyl-CoM
coenzyme B
methyl-coenzyme M reductase (Mt) : mrtA mrtB mrtG
methyl-coenzyme M reductase (Mt): mcrA mcrB mcrG
methyl-CoM reductase (Mt): mcrA mcrB mcrG
2-(methylthio)ethanesulfonate:N-(7-thioheptanoyl)-3-O-phosphothreonine S-(2-sulfoethyl)thiotransferase (Mb) : mcrA mcrB mcrG
2.8.4.1
methane

Supplementary Figure 6. **All methanogenesis pathways for the substrate bound fraction.** All identified proteins and proteins from only Archaea were mapped to these pathways. Presence of a heatmap ("heat block") beside an enzyme indicates presence, and the colors indicate fold change in log_2 units from 30 g/L. Order of the heatmap boxes – 30 g/L-75 g/L-120 g/L-150 g/L. Source data are provided as a Source Data file.

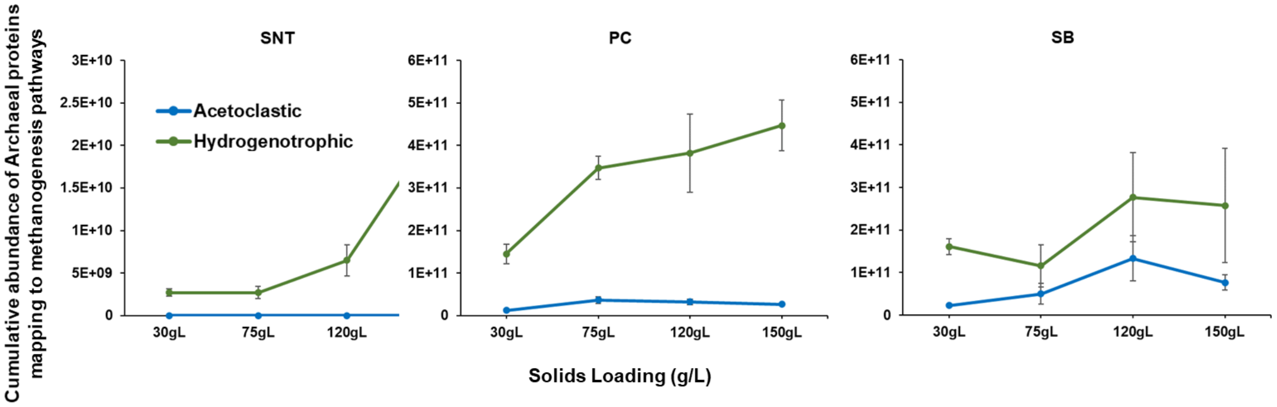

Supplementary Figure 7. **Summed abundance trends of the Archaeal proteins mapping to two major methanogenesis pathways**. It shows that hydrogenotrophic pathway is more abundant in each fraction and exhibits an increasing trend with solids loading. Data are presented as mean values ±SD (n=4 for 30 and 120 g/L, n=3 for 75 g/L, and n=5 for 150 g/L). SNT: Supernatant, PC: Planktonic cells, and SB: Substrate bound fraction. Source data are provided as a Source Data file.

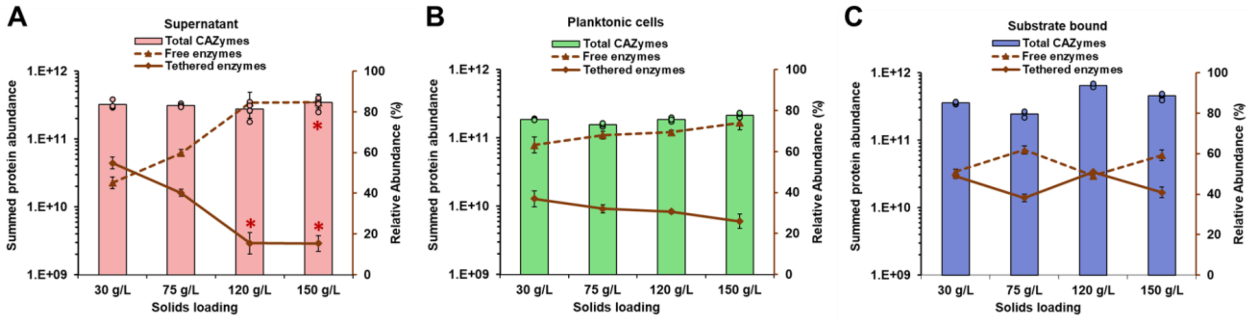

Supplementary Figure 8. **Proportion of tethered and free CAZymes as a function of solids loadings across fractions.** Summed abundance of microbial origin proteins mapping to different Carbohydrate Active enZymes (CAZymes) (**shown as bars**) and the relative contributions from the free and tethered (those with CBM/ cohesin/ dockerin) CAZymes (**shown as lines**) in the (**A**) Supernatant (SNT), (**B**) Planktonic cells (PC), and (**C**) Substrate bound (SB) fractions. Data are presented as mean values ±SD (n=4 for 30 and 120 g/L, n=3 for 75 g/L, and n=5 for 150 g/L). Overall, the number of free enzymes is greater than that of the tethered enzymes. As expected, the SB fraction has the highest proportion of its CAZymes' abundance coming from tethered enzymes compared to the other fractions. With change in solids loadings, in the three fractions, there appears to be partitioning between the free and tethered enzymes, which is especially evident in the supernatant fraction. The relative prevalence of free enzymes at the high solids suggest that they could be more important for solubilization. Source data are provided as a Source Data file.

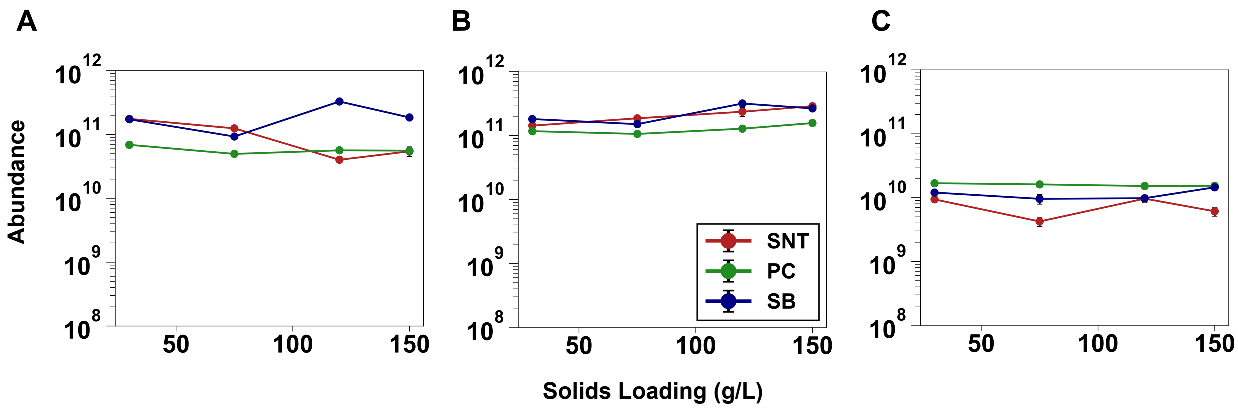

Supplementary Figure 9. **Total abundance patterns of select CAZyme subcategories with increasing solids loadings.** Summed abundance trends of proteins annotated as CAZymes (**A**) with one or more binding modules (CBM, dockerin, cohesin), (**B**) without any binding modules, and (**C**) with glycosyl transferase (GT) activity across the four switchgrass solids loadings in the three fractions. No significant change was seen in the cumulative abundances of GTs. SNT: Supernatant, PC: Planktonic cells, and SB: Substrate bound fraction. Source data are provided as a Source Data file.

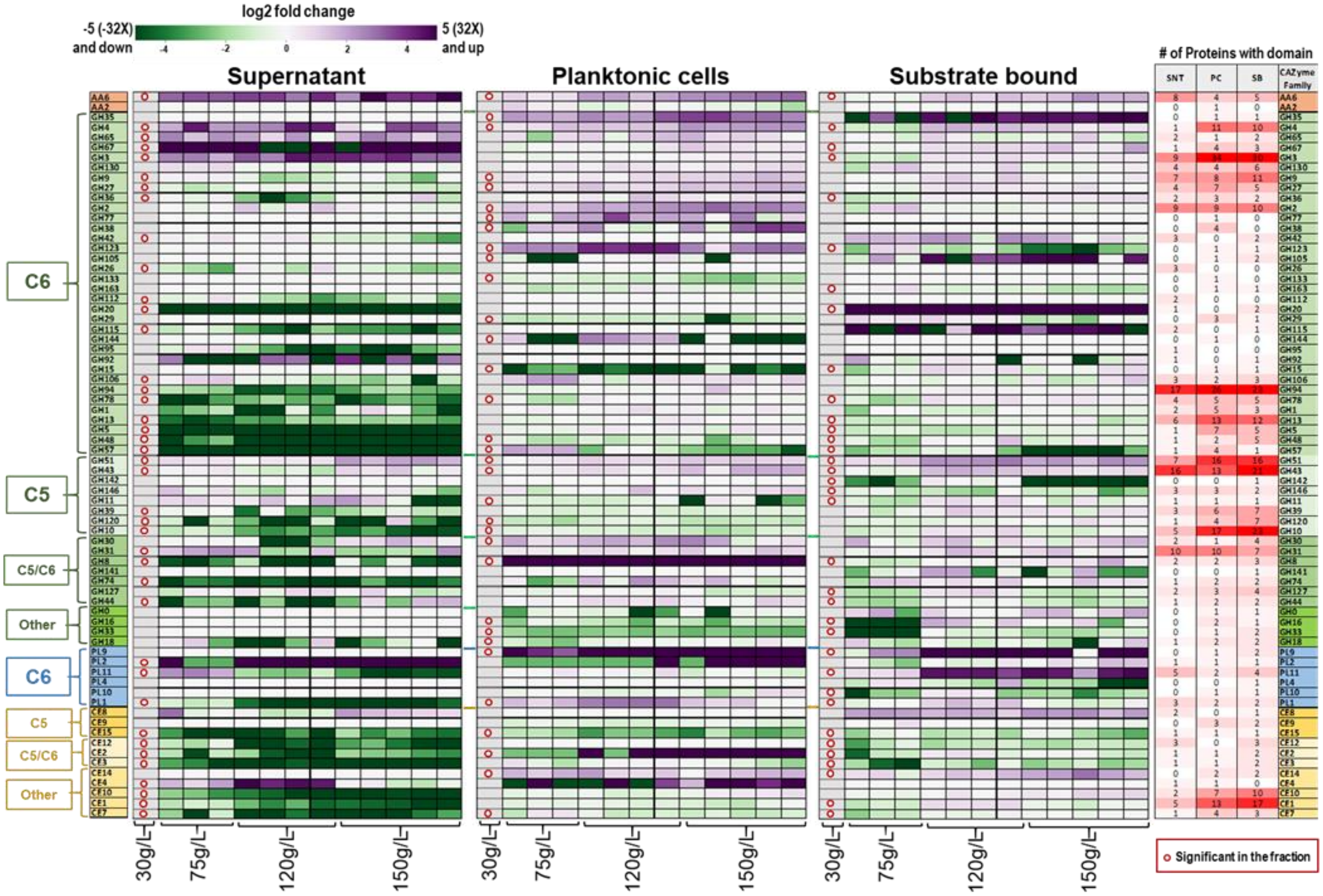

Supplementary Figure 10. **Fraction specific fold changes in all identified CAZyme families as a function of solids loadings.** Heatmap shows the abundance trend of proteins from CAZyme families that make up the hydrolytic CAZyme classes, grouped into potential C5/C6 activities based on the annotated function of the proteins that make up the respective family. On the right, the count of proteins that map that contain the respective CAZyme family domain are represented. A two-tailed Welch's *t*-test was performed from each solids loading versus the 30 g/L loading. Significant means *p*-value ≤0.05 and absolute $\log_2$ fold change ≥1 vs. 30 g/L condition. Source data are provided as a Source Data file.

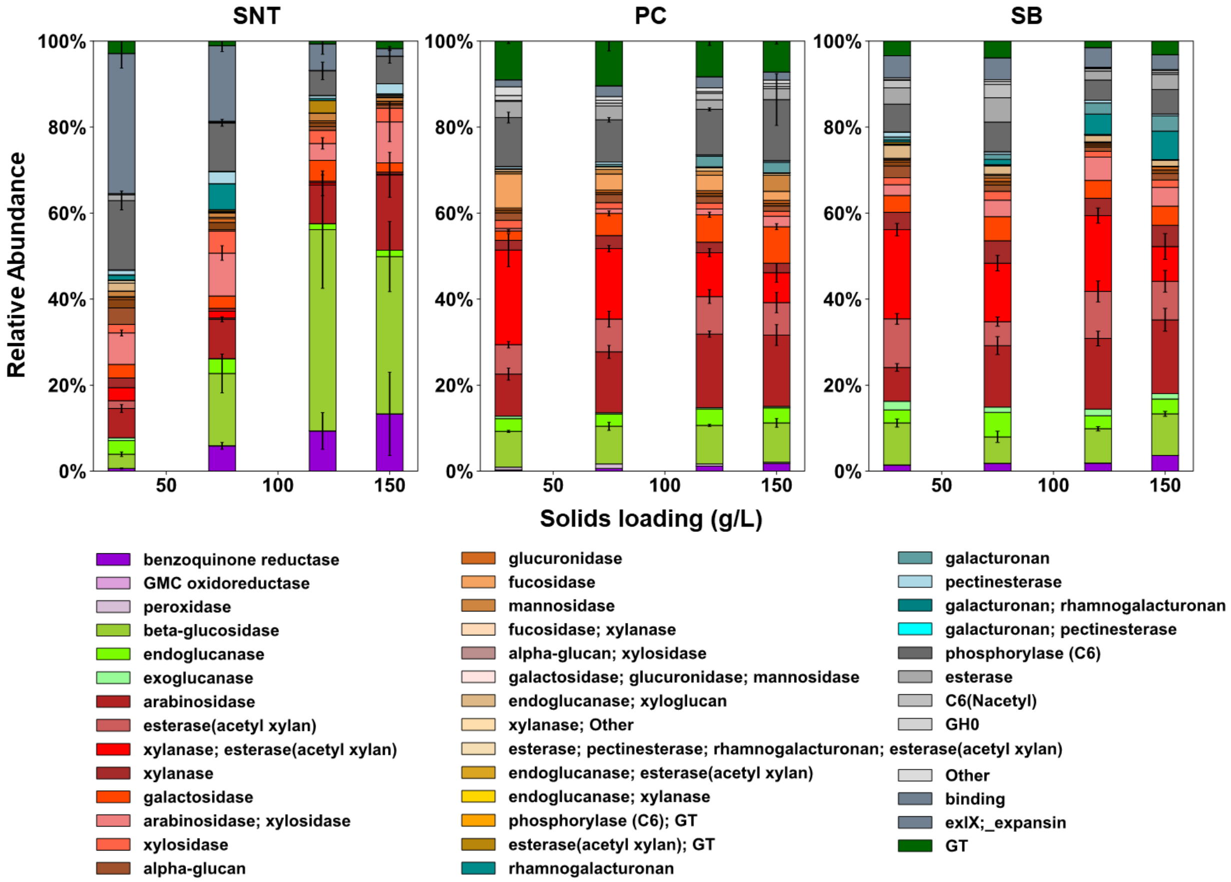

Supplementary Figure 11. **Proportion of enzymatic activities differ across fractions and with substrate loadings.** Abundance contributions of different substrate activities to the total CAZyme abundance in the three fractions- Supernatant (SNT), Planktonic cells (PC), and Substrate bound (SB) fraction across the four solids loadings. Here, each protein abundance has been accounted for only once in each fraction. Certain proteins had multiple domains and hence multiple activities, or their potential activity was indistinguishable based on annotation alone- in both these cases the proteins were considered to have all possible functions. Data are presented as mean values ±SD (n=4 for 30 and 120 g/L, n=3 for 75 g/L, and n=5 for 150 g/L). Error bars are shown for those categories with >=10% contribution to the total CAZyme intensity. Source data are provided as a Source Data file.

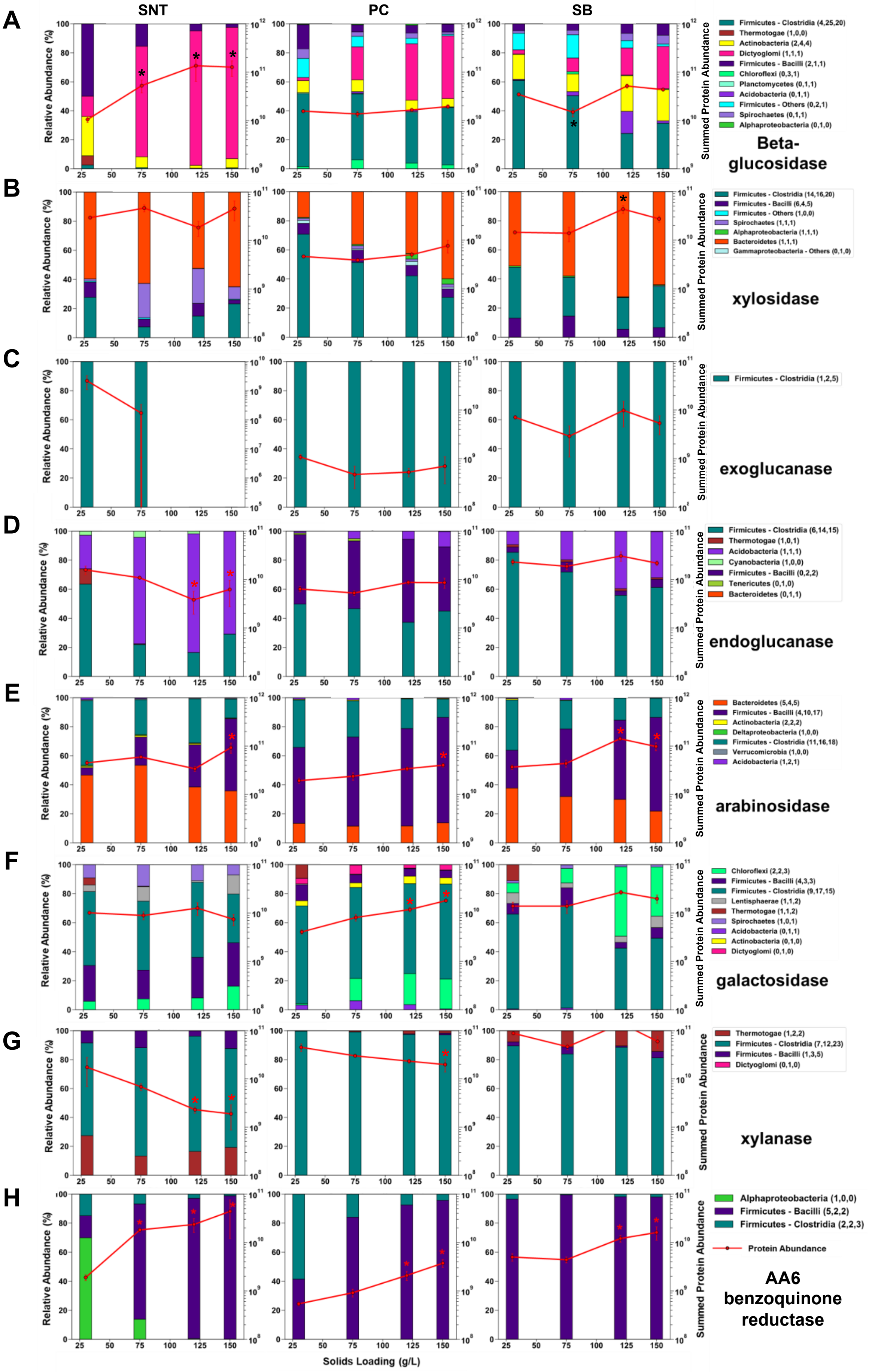

SNT
PC
SB
Relative Abundance (%)
Summed Protein Abundance
Solids Loading (g/L)
Firmicutes - Clostridia (4,25,20)
Thermotogae (1,0,0)
Actinobacteria (2,4,4)
Dictyoglomi (1,1,1)
Firmicutes - Bacilli (2,1,1)
Chloroflexi (0,3,1)
Planctomycetes (0,1,1)
Acidobacteria (0,1,1)
Firmicutes - Others (0,2,1)
Spirochaetes (0,1,1)
Alphaproteobacteria (0,1,0)
Beta-glucosidase
Firmicutes - Clostridia (14,16,20)
Firmicutes - Bacilli (6,4,5)
Firmicutes - Others (1,0,0)
Spirochaetes (1,1,1)
Alphaproteobacteria (1,1,1)
Bacteroidetes (1,1,1)
Gammaproteobacteria - Others (0,1,0)
xylosidase
Firmicutes - Clostridia (1,2,5)
exoglucanase
Firmicutes - Clostridia (6,14,15)
Thermotogae (1,0,1)
Acidobacteria (1,1,1)
Cyanobacteria (1,0,0)
Firmicutes - Bacilli (0,2,2)
Tenericutes (0,1,0)
Bacteroidetes (0,1,1)
endoglucanase
Bacteroidetes (5,4,5)
Firmicutes - Bacilli (4,10,17)
Actinobacteria (2,2,2)
Deltaproteobacteria (1,0,0)
Firmicutes - Clostridia (11,16,18)
Verrucomicrobia (1,0,0)
Acidobacteria (1,2,1)
arabinosidase
Chloroflexi (2,2,3)
Firmicutes - Bacilli (4,3,3)
Firmicutes - Clostridia (9,17,15)
Lentisphaerae (1,1,2)
Thermotogae (1,1,2)
Spirochaetes (1,0,1)
Acidobacteria (0,1,1)
Actinobacteria (0,1,0)
Dictyoglomi (0,1,0)
galactosidase
Thermotogae (1,2,2)
Firmicutes - Clostridia (7,12,23)
Firmicutes - Bacilli (1,3,5)
Dictyoglomi (0,1,0)
xylanase
Alphaproteobacteria (1,0,0)
Firmicutes - Bacilli (5,2,2)
Firmicutes - Clostridia (2,2,3)
Protein Abundance
AA6 benzoquinone reductase

Supplementary Figure 12. **Alterations in taxonomic drivers underlie total abundance changes in different enzymatic activities**. Phylum-resolved abundance trends of proteins annotated as (**A**) beta-glucosidase, (**B**) xylosidase, (**C**) exoglucanase, (**D**) endoglucanase, (**E**) arabinosidase, (**F**) galactosidase, (**G**) xylanase, and (**H**) AA6 (benzoquinone reductase). The numbers correspond to the count of proteins with the taxonomic origin identified for the respective activity in the three fractions – (SNT, PC, SB). A two-tailed Welch's *t*-test was performed with Benjamini Hochberg FDR correction from each solids loading versus the 30 g/L loading. * means *t*-test *p*-value ≤0.05 and absolute $\log_2$ fold change ≥1 vs. 30 g/L condition. Exact *P* values for each comparison are listed in Supplementary Data 6. Data are presented as mean values ±SD (n=4 for 30 and 120 g/L, n=3 for 75 g/L, and n=5 for 150 g/L). SNT: Supernatant, PC: Planktonic cells, and SB: Substrate bound fraction. Source data are provided as a Source Data file.

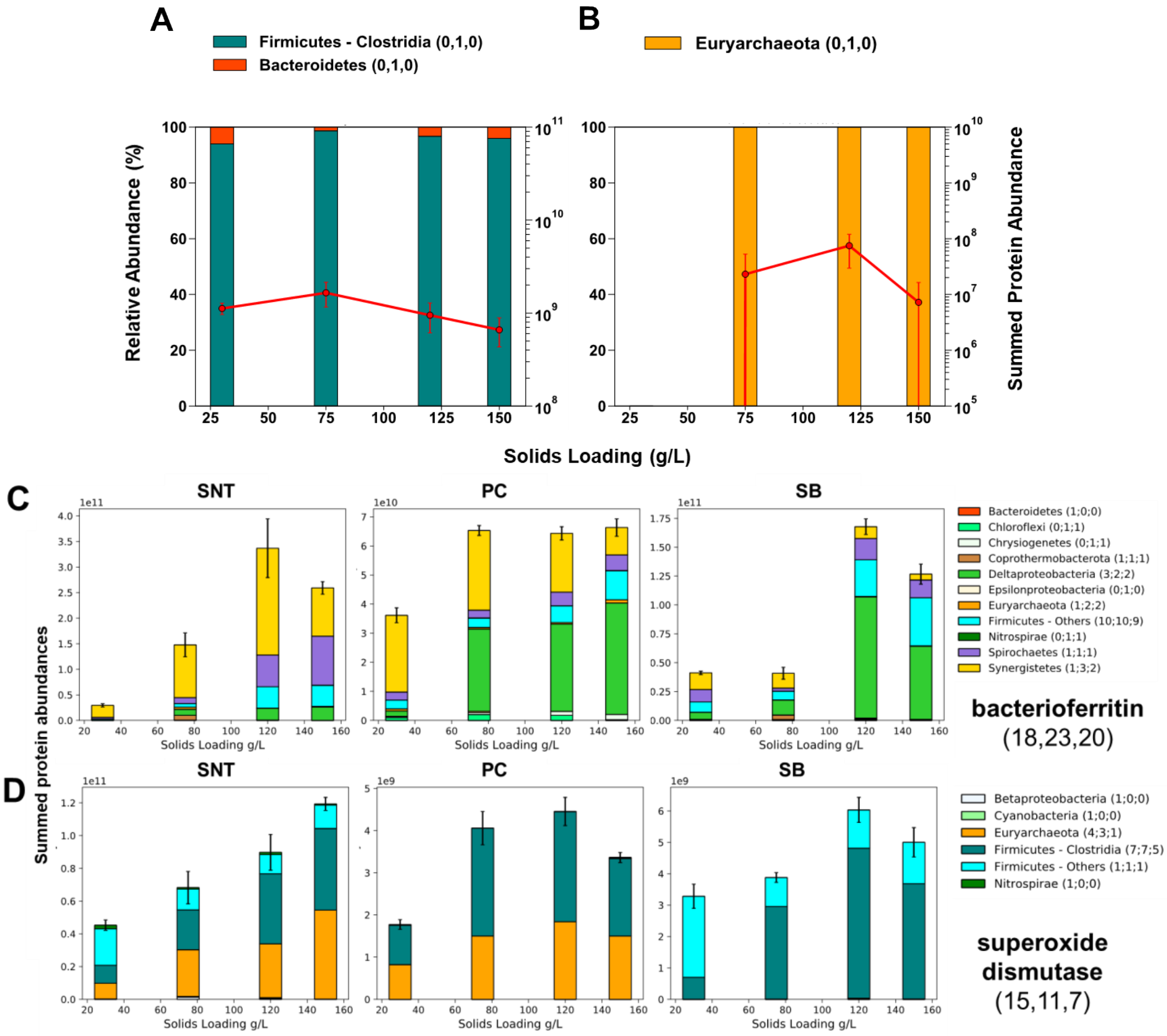

Supplementary Figure 13. **Multiple taxonomic groups contribute to lignin-acting and stress responsive enzymes.** Phylum-resolved abundance trends of proteins annotated as (**A**) AA2-Peroxidase (identified in PC fraction only), (**B**) AA3-GMC oxidoreductase (identified in PC fraction only), (**C**) Bacterioferritin, and (**D**) superoxide dismutase. The numbers correspond to the count of proteins identified with respective activity in the three fractions – (SNT, PC, SB). Data are presented as mean values ±SD (n=4 for 30 and 120 g/L, n=3 for 75 g/L, and n=5 for 150 g/L). SNT: Supernatant, PC: Planktonic cells, and SB: Substrate bound fraction. Source data are provided as a Source Data file.

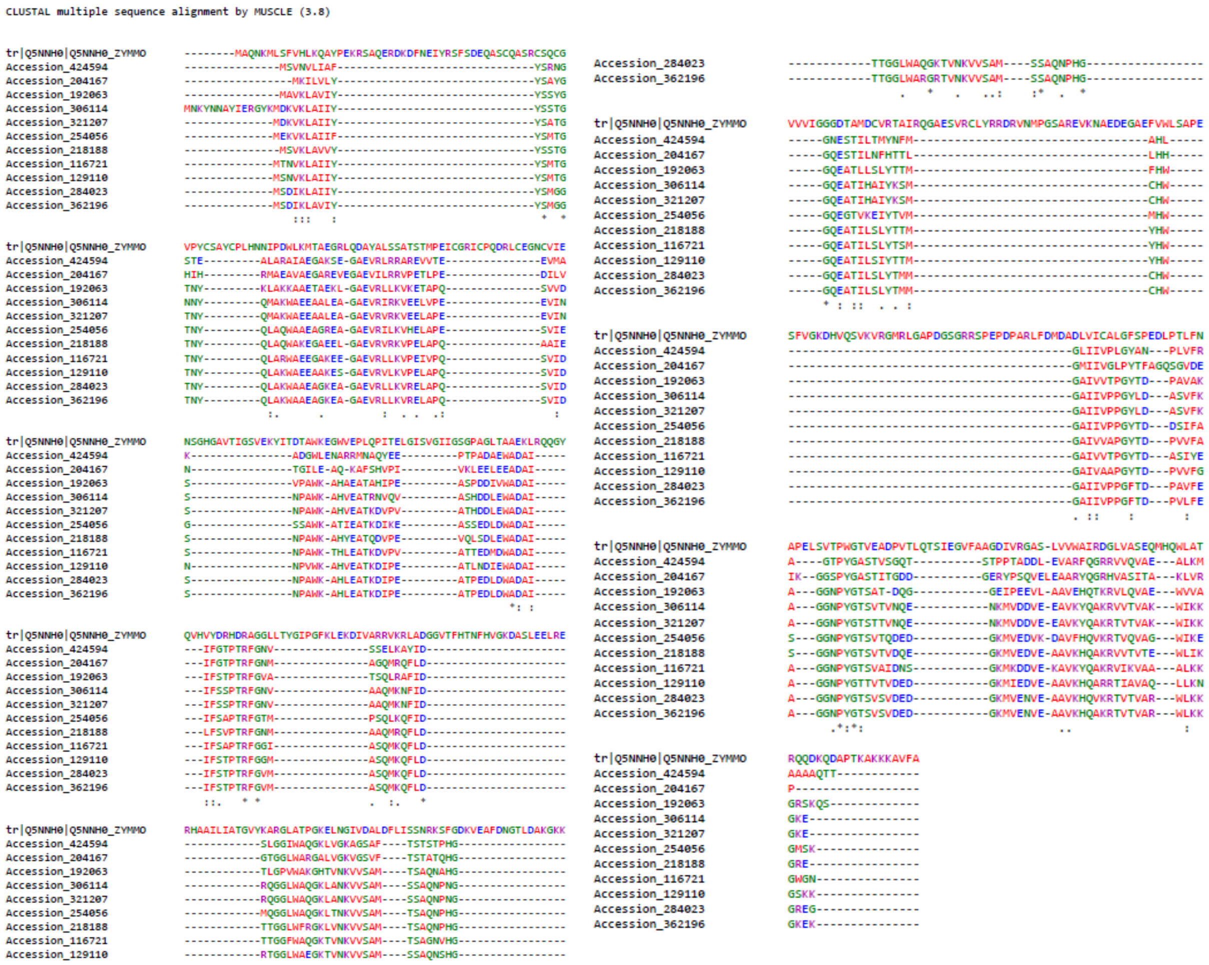

Supplementary Figure 14. Multiple sequence alignment of AA6 proteins against ZMO1116 using MUSCLE 3.8 program. Source data are provided as a Source Data file.

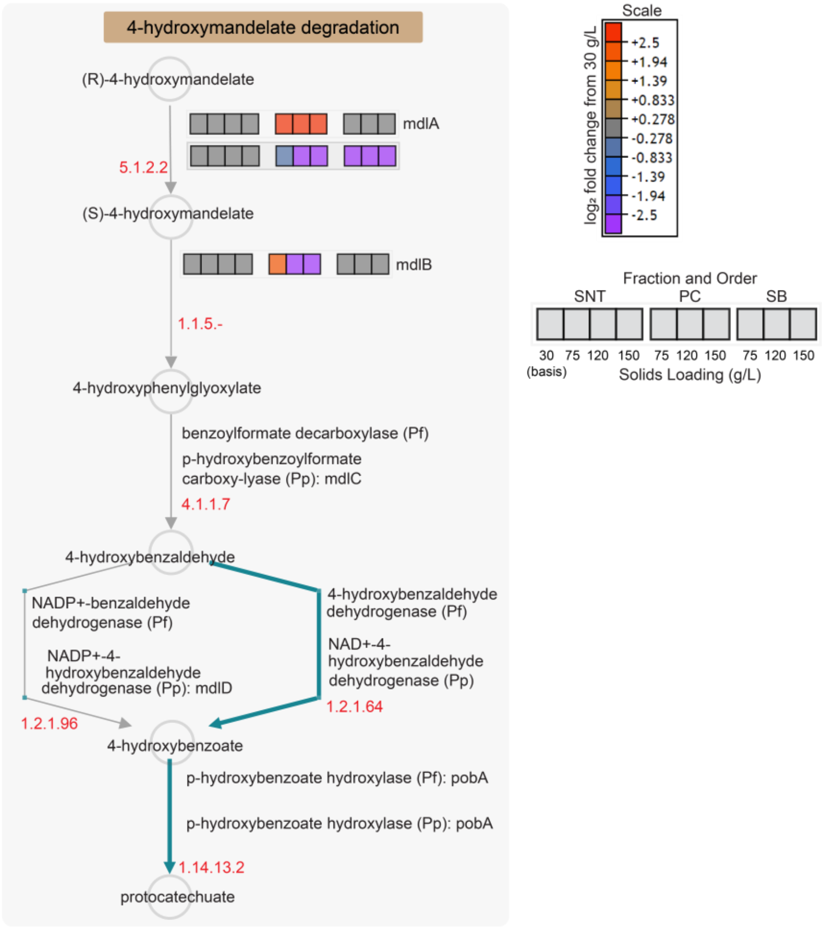

Supplementary Figure 15. 4-hydroxymandelate degradation pathway mapped with identified proteins. Presence is indicated by a heatmap beside the enzyme. Close examination revealed presence of very few proteins for most of those that were identified. Pathway diagram was generated using MetaCyc pathway collages. SNT: Supernatant, PC: Planktonic cells, and SB: Substrate bound fraction. Source data are provided as a Source Data file.

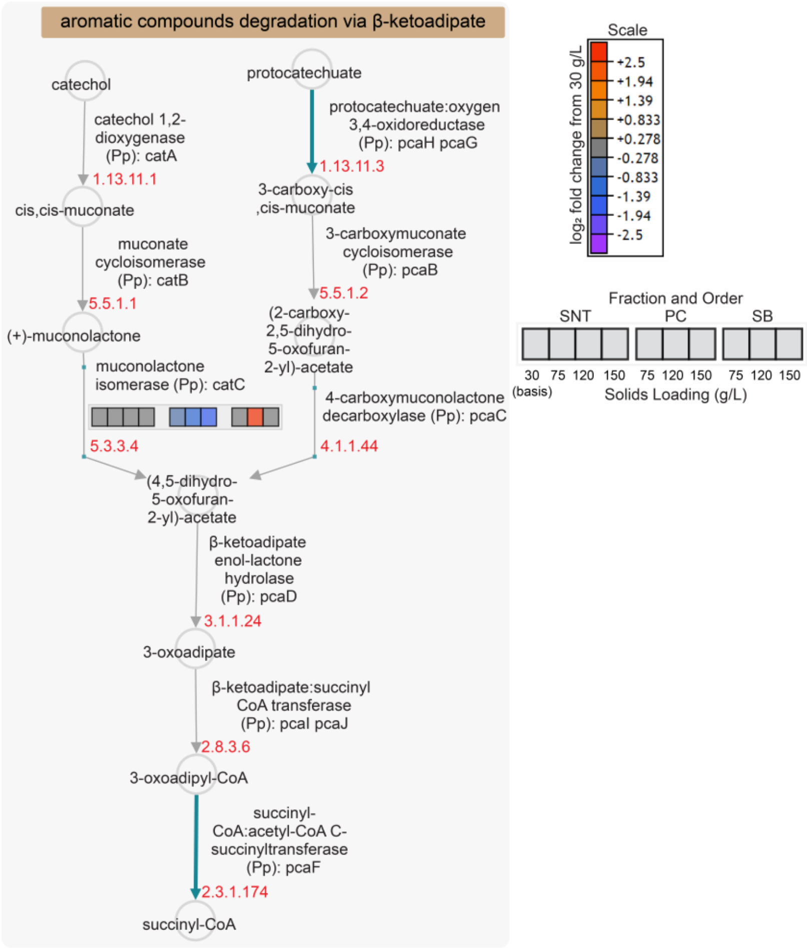

Supplementary Figure 16. Beta-ketoadipate pathway for degradation of aromatic compounds mapped with identified proteins. Presence is indicated by a heatmap beside the enzyme. Close examination revealed presence of very few proteins for most of those that were identified. Pathway diagram was generated using MetaCyc pathway collages. SNT: Supernatant, PC: Planktonic cells, and SB: Substrate bound fraction. Source data are provided as a Source Data file.

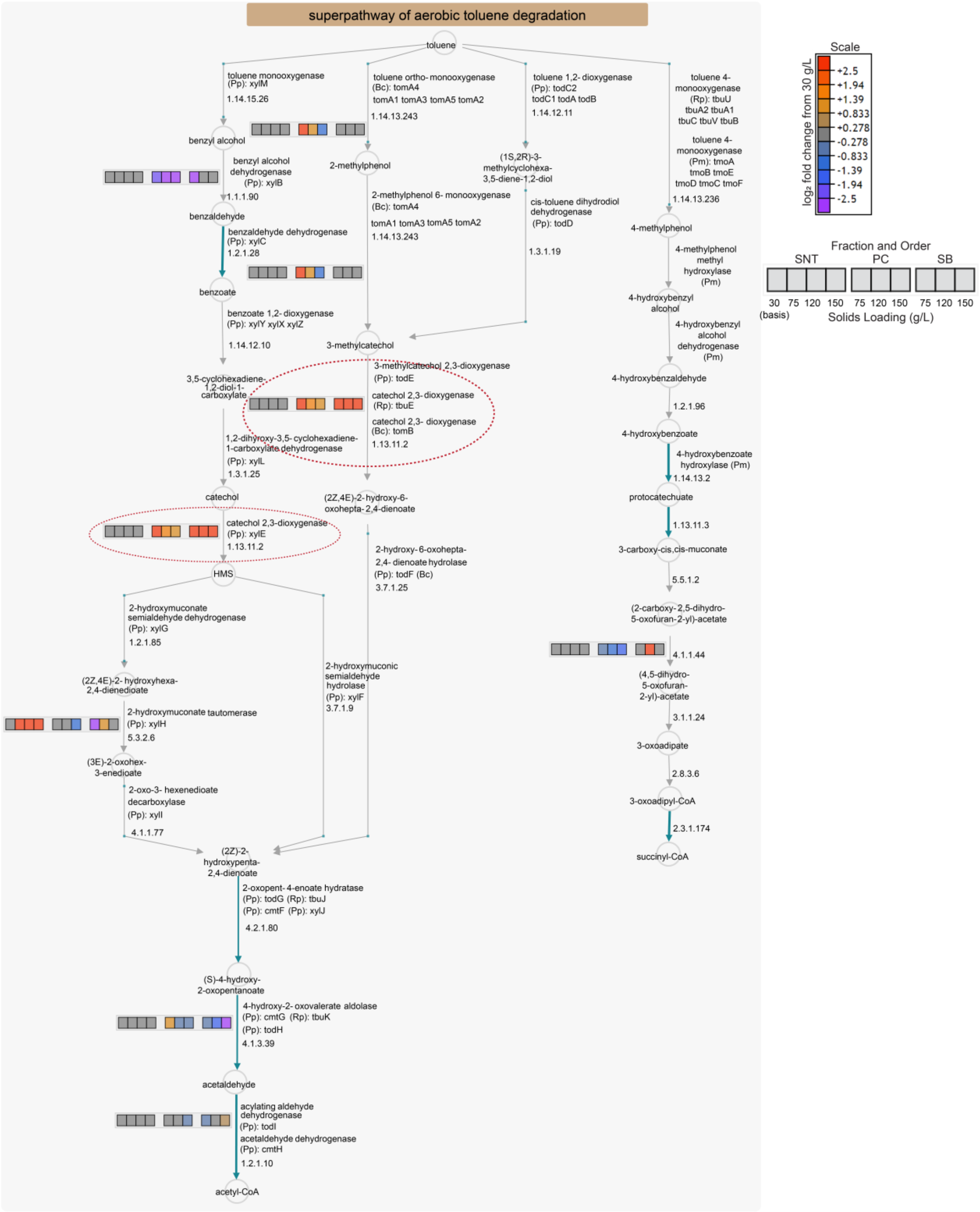

Supplementary Figure 17. Toluene degradation pathways mapped with identified proteins. Presence is indicated by a heatmap beside the enzyme. Close examination revealed presence of very few proteins for most of those that were identified. Additionally, those with increasing abundance seemed to be involved in catechol conversion as highlighted. Pathway diagram was generated using MetaCyc pathway collages. SNT: Supernatant, PC: Planktonic cells, and SB: Substrate bound fraction. Source data are provided as a Source Data file.

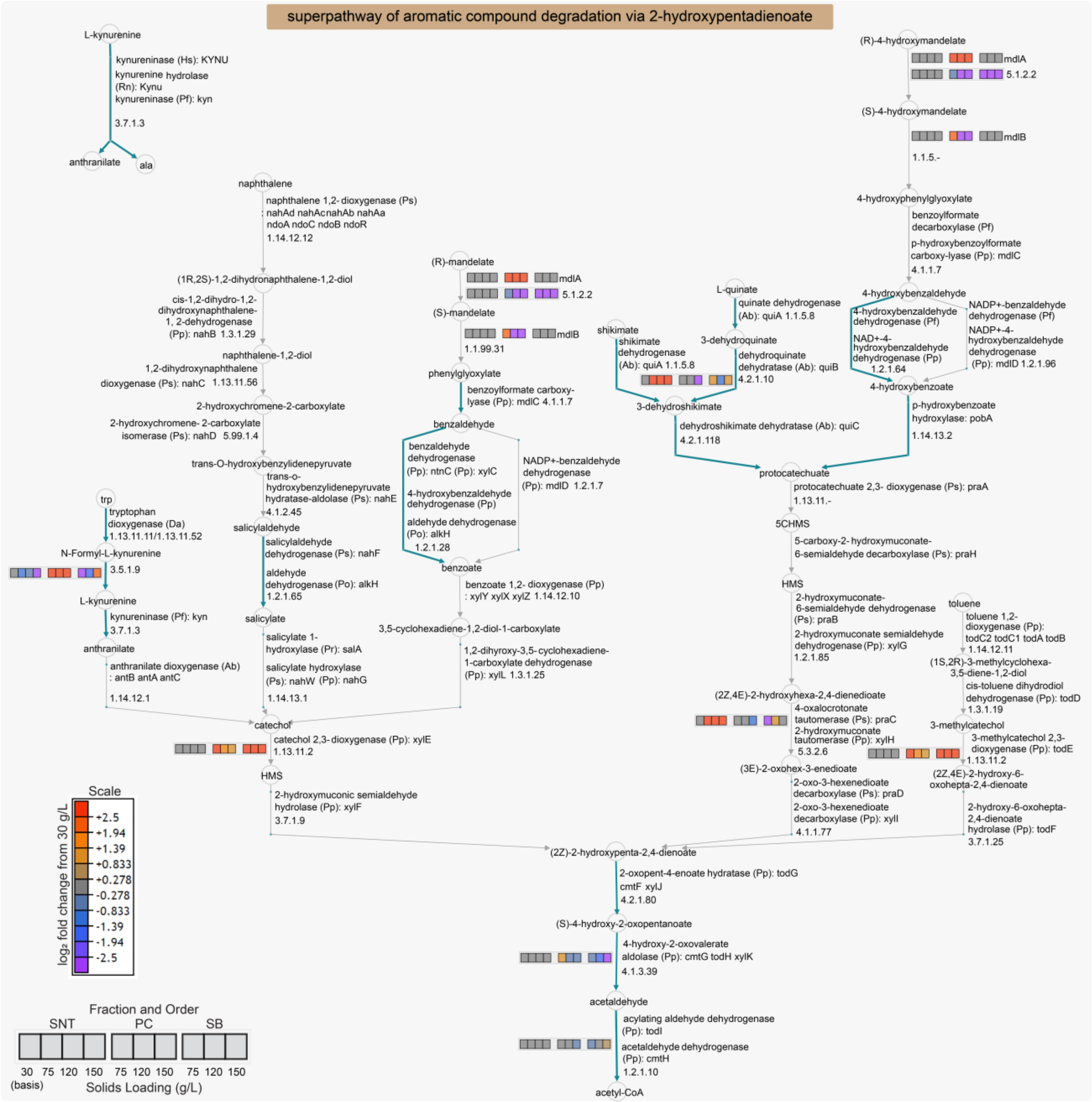

Supplementary Figure 18. Pathways for aromatic compound degradation via 2-hydroxypentadienoate mapped with identified proteins. Presence is indicated by a heatmap beside the enzyme. Close examination revealed presence of very few proteins for most of those that were identified. Pathway diagram was generated using MetaCyc pathway collages. SNT: Supernatant, PC: Planktonic cells, and SB: Substrate bound fraction. Source data are provided as a Source Data file.

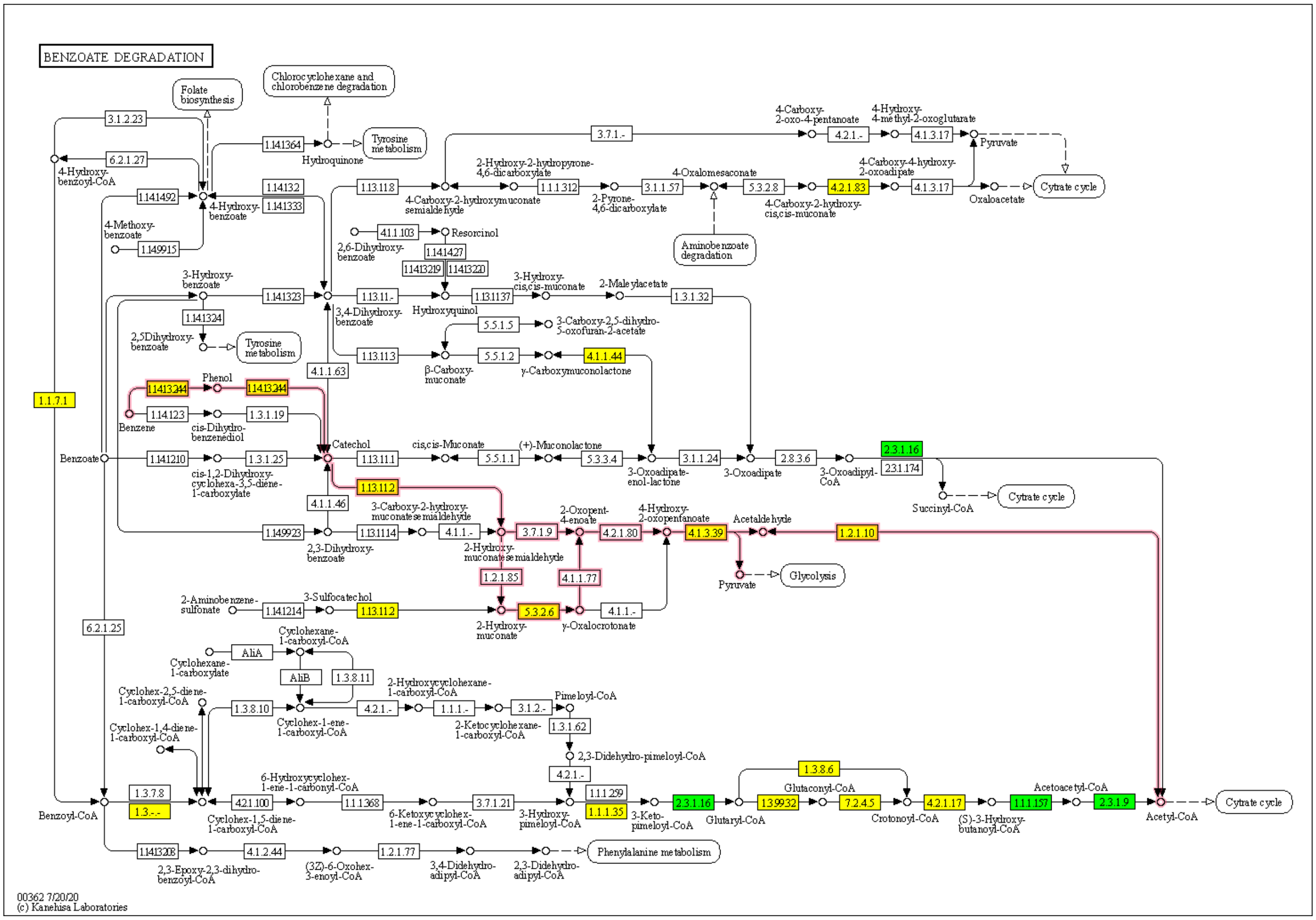

Supplementary Figure 19. Benzoate degradation pathway. The identified enzymes are colored (green or yellow- green were more numerous) and those highlighted in red constitute benzene degradation and Catechol meta-cleavage pathways. Pathway map was generated using KEGG pathways. Source data are provided as a Source Data file.

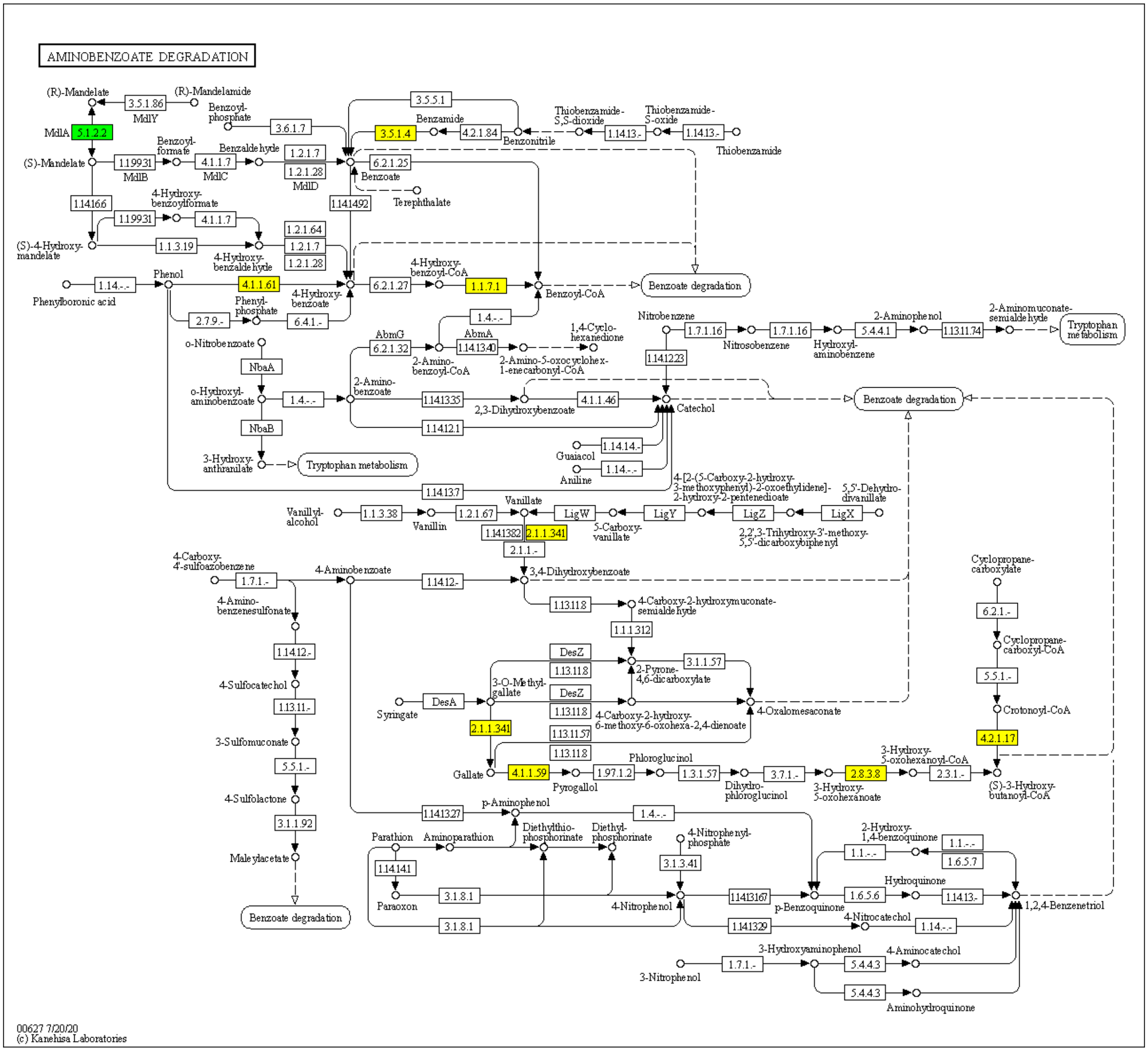

Supplementary Figure 20. Aminobenzoate degradation pathway with identified enzymes colored. Green or yellow- green were more numerous. Pathway map was generated using KEGG pathways. Source data are provided as a Source Data file.

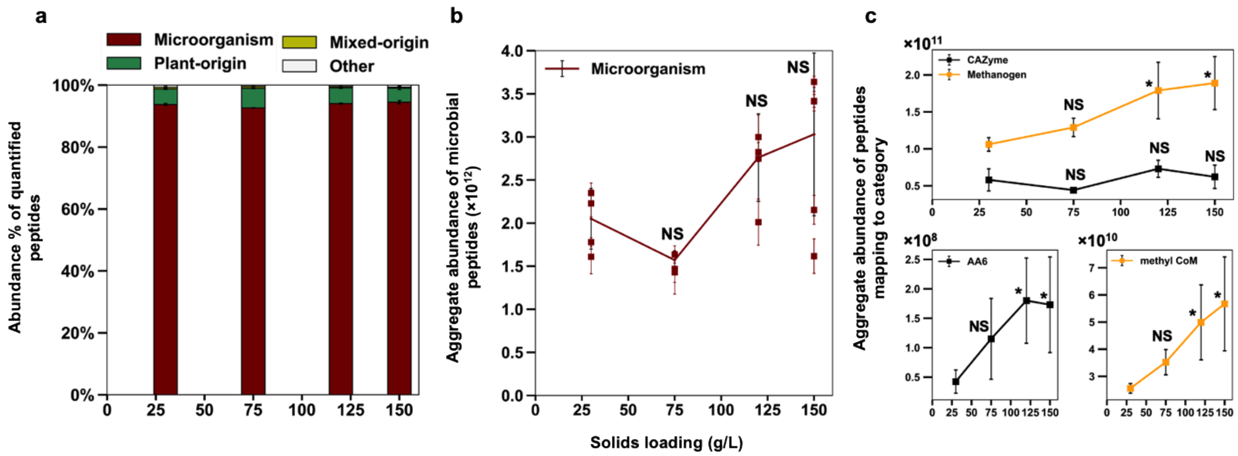

Supplementary Figure 21. **Total microbial biomass in the bioreactor with increasing solids loadings.** Microbial biomass was estimated by conducting a metaproteomic analysis on equal volumes of samples at each solids loading. (**a**) The resultant relative contribution of the different taxonomic categories to the total protein abundance at each solids loading. (**b**) The aggregate abundance of identified microbial peptides (considered proportional to microbial biomass) compared across the solids loadings. (**c**) The abundance trend of cellulolytic (and saccharolytic) and methanogenic organisms (inferred from aggregate abundance of identified CAZyme and *Euryarchaeota* peptides, respectively) (**top plot**), aggregate abundance trend of peptides mapping AA6 and methyl-coenzyme M proteins (**bottom left**) with solids loading. Error bars are ±standard deviation (n=4 for 30 and 120 g/L, n=3 for 75 g/L, and n=5 for 150 g/L with three methodological replicates for each). A two-tailed Welch's *t*-test was performed for each solids loading versus the 30 g/L loading. Presence of '*' means *t*-test *p*-value <0.05 vs. 30 g/L condition. Exact *P* values for each comparison are listed in Supplementary Data 1. **NS**, not significant; **CAZyme**, Carbohydrate Active enZyme. Source data are provided as a Source Data file.

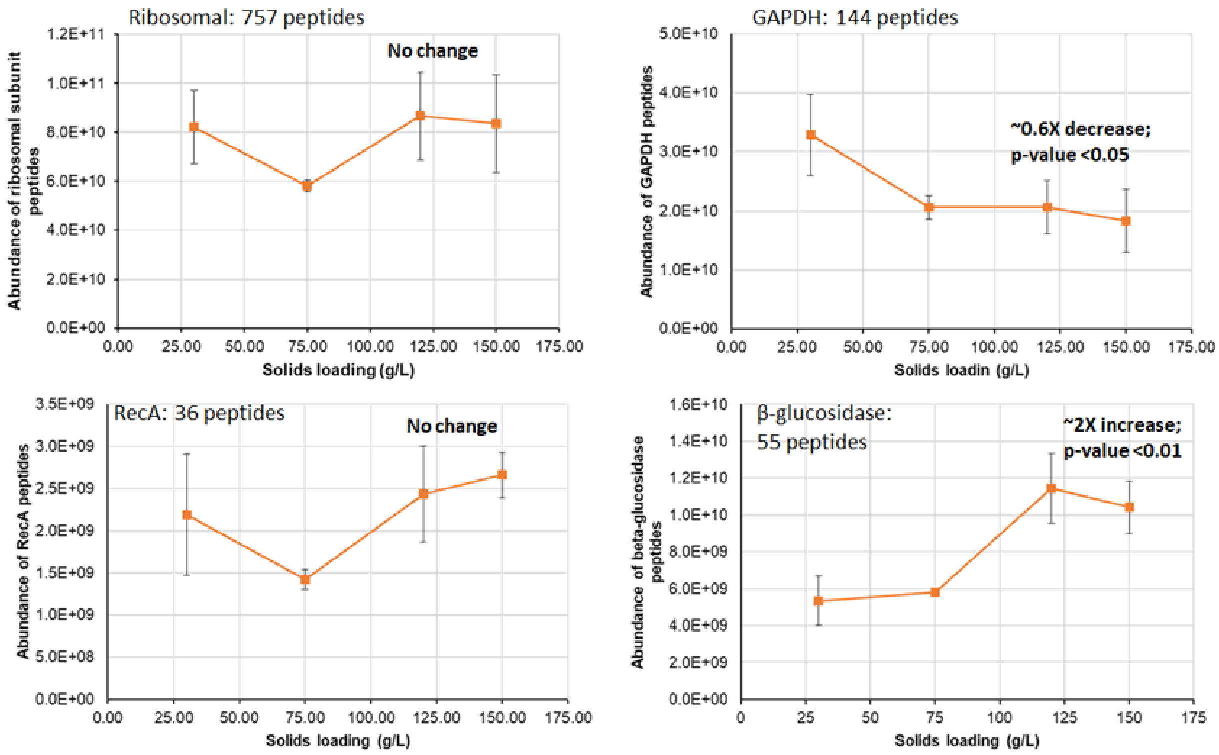

Supplementary Figure 22. Summed abundance trends of peptides from select housekeeping proteins based on the volume-normalized metaproteomic analysis. Error bars show ±standard deviation (n=4 for 30 and 120 g/L, n=3 for 75 g/L, and n=5 for 150 g/L with three methodological replicates for each). Source data are provided as a Source Data file.

Supplementary Note 1. Total microbial biomass in the bioreactor does not scale with the 5-fold increase in solid substrate loading

The observations above demonstrated that dynamic and customized tuning of the various lignocellulolytic enzymes and methanogenic pathways are clearly linked with sustained lignocellulosic deconstruction at high solids. However, a question remains about whether a change in total microbial biomass could also explain the solubilization results presented in Fig. 1B. Microbial cell biomass is usually limited by availability of substrates[2,3]; hence increased substrate concentrations can result in an equivalent proliferation in microbial cell biomass in the bioreactor (i.e., increased microbial density), which would also support the observation of undiminished solubilization at higher solids. Direct quantification of microbial biomass in the presence of solids is challenging as cells adhered to the plant substrate can complicate cell counting and optical density measurements; hence, microbial biomass can thus only be inferred based on indirect measurements[4]. For microbial communities, peptide and protein abundance data from metaproteomic measurements have been utilized to estimate the biomass amounts in the presence of solid substrates or otherwise murky environmental samples[5]. While traditional metaproteomic measurements provide detailed information about the diversity and relative abundance of proteins present in a fixed amount/mass of total protein extracted from a sample ("protein-normalized", as reported above), evaluation and comparison of total protein abundance obtained from a fixed volume of samples ("volume-normalized") is more relevant to estimate the concentration and yield of microbial biomass. In control experiments undertaken for this study, we demonstrated that such volume-normalized measurements positively correlate with microbial biomass for *C. thermocellum* and mock communities. Thus, a volume-normalized metaproteomic analysis was performed on unfractionated samples as a proxy to estimate microbial biomass changes across solids loadings.

To circumvent issues relating to shared peptides among orthologous proteins/domains, a peptide-centric approach was adopted to compare total peptide content. A total of 14,386 distinct peptides (highly confident, FDR ≤1%) were quantifiable across all samples. When peptides were mapped to kingdom of origin, we found that ~95% of peptide abundance was microbially-derived (Bacteria or Archaea), a metric that was consistent across the four solids loadings (Supplementary Fig. 21a, Supplementary Data 1). Given the increase in absolute carbohydrate solubilization rate from 30 g/L to 150 g/L (glucose, xylose, and arabinose), as well as a corresponding increase in methane production, microbial biomass would increase ~5-fold if

the cell yield per substrate consumed remained constant. However, we observed no significant change in total, volume-normalized microbial peptide abundance with increasing solids loading (Supplementary Fig. 21b), suggesting the microbial biomass (or cell-density) did not increase proportionately across the solids loadings conditions. This observation was further corroborated by the analysis of peptides from different housekeeping proteins which followed a similar trend (Supplementary Fig. 22) and showed no significant increase in aggregate abundance at higher solids loadings. Although the samples at 150 g/L demonstrated some biological variability, the increase in microbial biomass was still less than 2-fold for cultures with a 5-fold difference in substrate loading and substrate utilized. Thus, the rate of carbohydrate utilization normalized to total, volume-normalized microbial peptide abundance was more than twice as high in cultures with 150 g/L substrate loading as compared to cultures with 30 g/L substrate loading.

Since changes in community structure can still occur without changes in total amount[3], the abundance of select constituent subpopulations was further assessed at this unfractionated volume-normalized scale (Supplementary Fig. 21c, 22). This more quantitative viewpoint underscored and substantiated the observations made above: that no change in the total cellulolytic subpopulation (CAZyme) occurred while increases in specific enzymatic categories such as β-glucosidases and benzoquinone reductases (AA6) correlated with increased solids content. Furthermore, the aggregate abundance of peptides mapping to methanogens (*Euryarchaeota*) and methyl-coenzyme M reductase both maintained an increasing trend with solids loadings as was observed in the protein-normalized analysis presented earlier.

Supplementary Note 2. Methanogenesis primarily via the hydrogenotrophic pathway is observed in the microbiome

Enzymes associated with different methanogenesis pathways were explored across all fractions and solids loadings (Supplementary Fig. 3A, 4-6, Supplementary Data 5). The PC and SB fractions, which both include whole cells, had a greater representation of enzymes mapping to the different methanogenic pathways (Supplementary Fig. 3A). Although no proteins from methane oxidation were identified, complete (100%) pathway coverage was obtained for both acetoclastic and hydrogenotrophic methanogenesis in these two fractions (Supplementary Fig. 3A-B, 4-6). Enzymes from both pathways were generally represented by multiple proteins. Further analysis revealed that while the majority of enzymes associated with the hydrogenotrophic pathway were derived from *Euryarchaeota*, enzymes associated with the acetoclastic pathway also belonged to non-methanogenic bacteria. In an anaerobic community, bacterial members employ these enzymes to either produce acetate through the acetyl-CoA (Wood-Ljungdahl; WL) pathway or utilize acetate (via reversed WL pathway) as syntrophic acetate oxidizers to generate intermediates for the hydrogenotrophic pathway[6,7], so these were eliminated from methanogenesis consideration. Among the archaeal proteins, both the aggregate abundance and diversity (protein count) of those mapping the hydrogenotrophic pathway markedly exceeded that of the acetoclastic pathway (Supplementary Fig. 3A, 7), suggesting this to be the major route for methanogenesis. Coupled with syntrophic acetate oxidation for acetate degradation, hydrogenotrophic methanogenesis is the favored route under thermophilic temperatures[6,8] and has been observed previously in anaerobic thermophilic microbiomes[9]. At the level of individual reactions in the pathways, the abundance of archaeal enzymes from both methanogenic pathways increased uniformly with solids loadings, which was consistent with the measured increase in methane production (Fig. 1D, Supplementary Fig. 3B, 4-6).

All the methanogenesis pathway enzymes had a clearly increasing trend in the PC fraction which correlated with increasing solids loadings. This fraction also had the greatest representation of enzymes involved in the methanogenesis process (Supplementary Fig. 3A-B, 5). Closer inspection revealed that the PC fraction contained the highest total abundance of proteins from *Euryarchaeota* such that the phylum contributed a substantial portion of PC metaproteome compared to the other fractions (Supplementary Fig. 3C). This proportion increased from ~7% to ~14% from low to high solids loadings in the PC fraction, whereas it remained relatively steady in the other two fractions (SNT ~2% and SB ~5%) (Supplementary

Fig. 3C). These results indicate that this Archaea is primarily present in the PC fraction and is thus a cell-based or intracellular phenomenon that is not substrate-associated. Indeed, methanogenesis does not require proximity to lignocellulosic substrate but access to H_2 and CO_2, and the fractional observations made here, specifically its enrichment in non-substrate-associated planktonic cells, supports this interpretation.

Supplementary references

1 Liang, X. *et al.* Development and characterization of stable anaerobic thermophilic methanogenic microbiomes fermenting switchgrass at decreasing residence times. *Biotechnology for Biofuels* **11**, 1-18, doi:doi:10.1186/s13068-018-1238-1 (2018).

2 Baudoin, E., Benizri, E. & Guckert, A. Impact of artificial root exudates on the bacterial community structure in bulk soil and maize rhizosphere. *Soil Biol Biochem* **35**, 1183-1192, doi:10.1016/S0038-0717(03)00179-2 (2003).

3 Griffiths, B. S., Ritz, K., Ebblewhite, N. & Dobson, G. Soil microbial community structure: Effects of substrate loading rates. *Soil Biol Biochem* **31**, 145-153 (1999).

4 Lynd, L. R., Weimer, P. J., van Zyl, W. H. & Pretorius, I. S. Microbial cellulose utilization: Fundamentals and biotechnology. *Microbiol Mol Biol R* **66**, 506-+, doi:10.1128/Mmbr.66.3.506-577.2002 (2002).

5 Kleiner, M. *et al.* Assessing species biomass contributions in microbial communities via metaproteomics. *Nat Commun* **8**, 1558, doi:10.1038/s41467-017-01544-x (2017).

6 Timmers, P. H. A. *et al.* Metabolism and Occurrence of Methanogenic and Sulfate-Reducing Syntrophic Acetate Oxidizing Communities in Haloalkaline Environments. *Front Microbiol* **9**, 3039, doi:10.3389/fmicb.2018.03039 (2018).

7 Zhu, X. *et al.* Metabolic dependencies govern microbial syntrophies during methanogenesis in an anaerobic digestion ecosystem. *Microbiome* **8**, 22, doi:10.1186/s40168-019-0780-9 (2020).

8 Dyksma, S., Jansen, L. & Gallert, C. Syntrophic acetate oxidation replaces acetoclastic methanogenesis during thermophilic digestion of biowaste. *Microbiome* **8**, 105, doi:10.1186/s40168-020-00862-5 (2020).

9 Hagen, L. H. *et al.* Quantitative Metaproteomics Highlight the Metabolic Contributions of Uncultured Phylotypes in a Thermophilic Anaerobic Digester. *Appl Environ Microbiol* **83**, doi:10.1128/AEM.01955-16 (2017).
