# Supplementary material for: Metaproteomics reveals enzymatic strategies deployed by anaerobic microbiomes to maintain lignocellulose deconstruction at high solids
Source: Nat Commun. 2022 Jul 5;13:3870. doi: 10.1038/s41467-022-31433-x (PMC9256739; doi:10.1038/s41467-022-31433-x)
Supplement: Supplementary file 3 — Description Additional Supplementary Files [file 41467_2022_31433_MOESM3_ESM.pdf]

Description of Additional Supplementary Files

File Name: Supplementary Data 1
Description: All peptides quantified in the unfractionated volume-normalized metaproteomic analysis

File Name: Supplementary Data 2
Description: All proteins identified and quantified in the Supernatant (SNT) fraction

File Name: Supplementary Data 3
Description: All proteins identified and quantified in the Planktonic cells (PC) fraction

File Name: Supplementary Data 4
Description: All proteins identified and quantified in the Substrate bound (SB) fraction

File Name: Supplementary Data 5
Description: Methane Metabolism related enzymes quantified in the three fractions

File Name: Supplementary Data 6
Description: Carbohydrate Active Enzyme (CAZyme) proteins identified and quantified in all three (SNT, PC, SB) fractions

File Name: Supplementary Data 7
Description: Substrate activity grouping of the identified CAZyme proteins in the three (SNT, PC, SB) fractions.

File Name: Supplementary Data 8
Description: Measured proteins with potential oxidase activity based on KEGG Orthology (KO) in each fraction.

File Name: Supplementary Data 9
Description: Reactive Oxygen Species (ROS) stress responsive enzymes
