# Supplementary material for: Metaproteomics reveals enzymatic strategies deployed by anaerobic microbiomes to maintain lignocellulose deconstruction at high solids
Source: Nat Commun. 2022 Jul 5;13:3870. doi: 10.1038/s41467-022-31433-x (PMC9256739; doi:10.1038/s41467-022-31433-x)
Supplement: Supplementary file 13 — Reporting Summary [file 41467_2022_31433_MOESM13_ESM.pdf]

nature portfolio

Corresponding author(s): Lee R. Lynd, Robert L. Hettich

Last updated by author(s): Jun 2, 2022

Reporting Summary

Nature Portfolio wishes to improve the reproducibility of the work that we publish. This form provides structure for consistency and transparency in reporting. For further information on Nature Portfolio policies, see our Editorial Policies and the Editorial Policy Checklist.

Statistics

For all statistical analyses, confirm that the following items are present in the figure legend, table legend, main text, or Methods section.

n/a	Confirmed	
☐	☒	The exact sample size (*n*) for each experimental group/condition, given as a discrete number and unit of measurement
☐	☒	A statement on whether measurements were taken from distinct samples or whether the same sample was measured repeatedly
☐	☒	The statistical test(s) used AND whether they are one- or two-sided<br>*Only common tests should be described solely by name; describe more complex techniques in the Methods section.*
☒	☐	A description of all covariates tested
☐	☒	A description of any assumptions or corrections, such as tests of normality and adjustment for multiple comparisons
☐	☒	A full description of the statistical parameters including central tendency (e.g. means) or other basic estimates (e.g. regression coefficient) AND variation (e.g. standard deviation) or associated estimates of uncertainty (e.g. confidence intervals)
☐	☒	For null hypothesis testing, the test statistic (e.g. *F*, *t*, *r*) with confidence intervals, effect sizes, degrees of freedom and *P* value noted<br>*Give P values as exact values whenever suitable.*
☒	☐	For Bayesian analysis, information on the choice of priors and Markov chain Monte Carlo settings
☒	☐	For hierarchical and complex designs, identification of the appropriate level for tests and full reporting of outcomes
☐	☒	Estimates of effect sizes (e.g. Cohen's *d*, Pearson's *r*), indicating how they were calculated

Our web collection on statistics for biologists contains articles on many of the points above.

Software and code

Policy information about availability of computer code

Data collection	Primary fermentation data (pH, Temperature, Stirrer rpm, pH controlled base addition) was recorded using Module Operator Service Program (MFCS) data collection software (3.0, level 43, 2008 Sartorius Stedim Systems). LC-MS/MS data was collected on a Q Exactive Plus mass spectrometer running Xcalibur (v.4.2.47; Thermo Scientific) data collection software. MS/MS data were searched using Proteome Discoverer (v.2.3; Thermo Scientific) software as described in Materials and Methods.
Data analysis	The program Veusz (v3.4.01, https://veusz.github.io/) was used for visualization of primary and secondary fermentation data.<br>Metagenomic sequences were assembled using MEGAHIT (Bioinformatics (2015) doi:10.1093/bioinformatics/btv033) and genes identified with Prodigal (BMC Bioinformatics (2010) doi:10.1186/1471-2105-11-119). Protein abundance data was transformed and normalized with InfernoRDN (Bioinformatics (2008) doi: 10.1093/bioinformatics/btn217). Imputation and statistical analysis of proteomics data was performed in Perseus (Nature Methods (2016) doi:10.1038/nmeth.3901). Annotation of proteins was performed with KEGG GhostKOALA (Journal of Molecular Biology (2016) doi:10.1016/j.jmb.2015.11.006), eggNOG-mapper (Nucleic Acids Research (2019) doi: 10.1093/nar/gky1085), MetaCyc (Nucleic Acids Research (2018) doi:10.1093/nar/gkx935), dbCAN2 (Nucleic Acids Research (2018) doi: 10.1093/nar/gky418) amd InterProScan(Nucleic Acids Research (2020) doi: 10.1093/nar/gkaa977). CheckM (Genome Research (2015) doi: 10.1101/gr.186072.114) and GTDB-Tk (Bioinformatics (2020) doi: 10.1093/bioinformatics/btz848) were used for additional taxonomic annotations. Microsoft Excel, BioVenn (BMC Genomics (2008) doi.org/10.1186/1471-2164-9-488), BioRender, Python (NumPy, Pandas, Seaborn, Matplotlib), and R packages were used for data parsing and visualization. MUSCLE (Nucleic Acids Research (2004) doi.org/10.1093/nar/gkh340), MAFFT(Nucleic Acids Research (2002) doi.org/10.1093/nar/gkf436), and ClustalOmega (Protein Science (2018) doi: 10.1002/pro.3290) were used for performing protein multiple sequence alignments.

For manuscripts utilizing custom algorithms or software that are central to the research but not yet described in published literature, software must be made available to editors and reviewers. We strongly encourage code deposition in a community repository (e.g. GitHub). See the Nature Portfolio guidelines for submitting code & software for further information.

Data

Policy information about availability of data

All manuscripts must include a data availability statement. This statement should provide the following information, where applicable:

- Accession codes, unique identifiers, or web links for publicly available datasets
- A description of any restrictions on data availability
- For clinical datasets or third party data, please ensure that the statement adheres to our policy

Data on switchgrass solubilization and microbiome performance is available in supplementary information files. All proteome abundance data are available as supplementary data files. All raw mass spectra used for protein quantification is available at the ProteomeXchange Consortium via the MassIVE repository (massive.ucsd.edu/) MassIVE accession: MSV000088319; ProteomeXchange accession: PXD029582. The data has been made publicly available. Source data has been provided with the manuscript.

Field-specific reporting

Please select the one below that is the best fit for your research. If you are not sure, read the appropriate sections before making your selection.

[x] Life sciences [ ] Behavioural & social sciences [ ] Ecological, evolutionary & environmental sciences

For a reference copy of the document with all sections, see nature.com/documents/nr-reporting-summary-flat.pdf

Life sciences study design

All studies must disclose on these points even when the disclosure is negative.

Sample size	All sample sizes used are listed in the manuscript and also indicated in the supplemental Table S2. For fermentation related sampling, keeper data was only considered after at least 3 residence times and the system to be in steady state as witnessed by solubilization, gas volumetric output, and methane concentration results. Samples used for further data analysis as shown in this manuscript were taken for at least 2 residence times after reaching steady state, after which the system was changed to a new solids loading. Primary data such as solubilization and methane concentration were obtained via physical material sampling, off-gas rate data was obtained via sampling on-line data once per day, both determined the sample size. For proteomic measurements, no sample size calculation was performed. Sample size (n>=3) represents field standard and is regular practice in literature.
Data exclusions	No data was excluded for proteomics analysis.
Replication	As shown in tables S1, S2, and figure S1 - fermentation samples were collected at steady state conditions, and analysis was performed for the number of samples indicated. All attempts at replication were successful and reproducibility is built in to the steady-state replicate samples measured in this study. Key findings were confirmed through observations from multiple proteins across multiple runs.
Randomization	Samples for proteomics were not run by LC-MS/MS in any particular order, and were ambiguously coded before the measurements. After the runs, a look-up table was used to identify experimental groups based on solids loading. Additionally, the proteomes were measured stochastically by the mass spectrometer via data-dependent acquisition and not targeted to any specific protein or protein class. On the proteome level, the samples clustered based on their experimental groups for all three fractions as well as whole broth samples. Fermentation related data randomization was not relevant due to a microbiome culture being studied.
Blinding	Proteomic measurements were inherently unbiased in that distributions of biomolecules (peptides/proteins) were measured. For fermentation related data blinding was not relevant due to one microbiome culture being sampled, which is unbiased and does not need blinds.

Reporting for specific materials, systems and methods

We require information from authors about some types of materials, experimental systems and methods used in many studies. Here, indicate whether each material, system or method listed is relevant to your study. If you are not sure if a list item applies to your research, read the appropriate section before selecting a response.

Materials & experimental systems

n/a	Involved in the study
[x]	[ ] Antibodies
[x]	[ ] Eukaryotic cell lines
[x]	[ ] Palaeontology and archaeology
[x]	[ ] Animals and other organisms
[x]	[ ] Human research participants
[x]	[ ] Clinical data
[x]	[ ] Dual use research of concern

Methods

n/a	Involved in the study
[x]	[ ] ChIP-seq
[x]	[ ] Flow cytometry
[x]	[ ] MRI-based neuroimaging
